# Neural mechanism of trigeminal nerve stimulation recovering defensive arousal responses in traumatic brain injury

**DOI:** 10.7150/thno.106323

**Published:** 2025-01-13

**Authors:** Qian Zhang, Haiyun Ma, Lifang Huo, Shaoling Wang, Qian Yang, Zhimin Ye, Jie Cao, Shaoling Wu, Chao Ma, Congping Shang

**Affiliations:** 1 Department of Rehabilitation, Sun Yat-sen Memorial Hospital, Sun Yat-sen University, Guangzhou 510030, China.; 2 School of Basic Medical Sciences, Guangzhou National Laboratory, The Fifth Affiliated Hospital, Guangzhou Medical University, Guangzhou 510005, China.

**Keywords:** Trigeminal nerve stimulation, Traumatic brain injury, Defensive arousal responses, Visual receptive field sensitivity, TG neuronal subgroups

## Abstract

The arousal state is defined as the degree to which an individual is aware of themselves and their surroundings, and is a crucial component of consciousness. Trigeminal nerve stimulation (TNS), a non-invasive clinical neuromodulation technique, has shown potential in aiding the functional recovery of patients with impaired consciousness. Understanding the specific neuronal subpopulations and circuits through which TNS improves arousal states is essential for advancing its clinical application.

**Methods:** A mouse model of traumatic brain injury (TBI) was established using a weight-drop technique to induce neurological dysfunction, and the arousal state was assessed through visual and auditory defensive responses. Techniques such as viral tracing, chemogenetics, patch-clamp recordings, calcium signaling, and neurotransmitter probes were employed to investigate the relevant subpopulations of trigeminal ganglion (TG) neurons and the underlying mechanisms in the central nervous system.

**Results:** Neuronal subgroups involved in TNS therapy at the key peripheral nucleus, the TG, were identified. Two distinct types of neurons were found to contribute differently: The Tac1+TG-locus coeruleus (LC)-superior colliculus (SC) pathway elevated noradrenaline levels in the SC, enhancing receptive field sensitivity recovery in TBI mice; the Piezo2+TG-paraventricular hypothalamic nucleus (PVN)-substantia nigra pars compacta (SNc)-dorsal striatum (DS) pathway initiated dopamine (DA) release in the DS, ameliorating motor disorders in TBI mice.

**Conclusion:** These pathways contribute to the improvement of defensive arousal responses from different perspectives. The findings from this study imply that TNS effectively restores defensive arousal responses to visual and auditory threats in mice suffering from TBI, offering insights that may facilitate the implementation of TNS therapy in clinical settings.

## Introduction

Impairment of the arousal state is prevalent among patients with traumatic brain injury (TBI), affecting millions globally each year and potentially leading to short-term or lifelong disabilities 1. Patients exhibiting impaired arousal states demonstrate reduced or lost abilities to recognize their own condition and respond to external stimuli 2. The recovery process from various coma levels involves cortical activity and behavioral restoration, marked by a transition from lower to higher arousal levels 3. This transition is characterized by a progressive improvement in the perception of external stimuli and self-awareness, encompassing language, vision, and motor functions. In clinical settings, the trajectory of arousal state recovery remains unpredictable; it may unfold over weeks or years, and some patients may never regain full consciousness. This variability presents a significant challenge, imposing significant burdens on patients, their families, and society. Consequently, the development of effective treatments that can rehabilitate patients' perception of external stimuli and elevate their arousal levels is of paramount importance.

To explore the internal states of arousal in experimental subjects 4, defensive arousal or alertness levels are inferred from their response rates and reaction times to sensory stimuli 5, 6. Innate defensive behaviors, essential for animal survival, are triggered by perceived environmental threats and can be elicited through visual and auditory stimuli 7. Employing this behavioral paradigm enables the establishment of a descriptive framework that effectively assesses the reactive capabilities of the subjects 8.

When confronted with a visual threat, the retina and superior colliculus (SC) initially perform stimulus detection and threat recognition. Subsequently, various subcortical regions involved in determining the freeze or flight response are activated, culminating in the brainstem producing the corresponding behavior 9. Visual sensing neurons encode two critical parameters: object size and approach speed, both of which converge on the superficial layer of the SC (sSC). Within the sSC, two distinct groups of neurons orchestrate escape and freezing behaviors: wide-field cells in the SC project to the lateral posterior nucleus (LP) to induce freezing, whereas parvalbumin+ (PV+) neurons project to the parabigeminal nucleus (PBG) to initiate escape behaviors 10. The intermediate and deep layers of the SC (m/dSC) facilitate sensory-motor conversion, projecting to multiple thalamic nuclei and the spinal cord to elicit a rapid response. This defense pathway involves numerous nuclei 11.

In contrast to the visual system, innate defense circuits in the auditory system have received less attention. The inferior colliculus (IC) plays a pivotal role in sound-driven innate defense behavior. Corticofugal neurons in the auditory cortex (ACx) target the IC, mediating an innate, sound-induced flight behavior 12. Neurons in the striatum and colliculus also contribute to this process 13.

In clinical practice, it has been observed that trigeminal nerve stimulation (TNS), a non-invasive transcranial electrical stimulation therapy 14, can induce awakening in patients with long-term disorders of consciousness (DOC) 15. Additionally, clinical randomized double-blind controlled trials have demonstrated that TNS facilitates functional recovery in patients with prolonged consciousness disorders 16. Rhythmic acoustic-electric trigeminal nerve stimulation, where musical stimulation is synchronized with electrical stimulation of the trigeminal nerve in the gamma band, has been shown to improve consciousness in patients with DOC 17. Furthermore, evidence indicates that activation of the trigeminal nerve system provides neuroprotection following brain damage 18. Preliminary experiments have demonstrated that TNS can activate the trigeminal ganglion (TG) 19, which is located at the peripheral input end. However, there has been no definitive conclusion regarding the specific subgroups of TG neurons involved in TNS treatment for TBI mice, nor about whether different subgroups have distinct neural projection pathways. Therefore, this study aimed to investigate the impact of TNS on the level of defensive arousal following TBI, as well as the neural circuits mediated by different TG neuron subgroups, to aid in understanding how TNS could be beneficial in treating arousal state impairment following TBI.

## Results

### TNS improves defensive arousal responses and motor abilities in TBI mice

**Figure 1:**
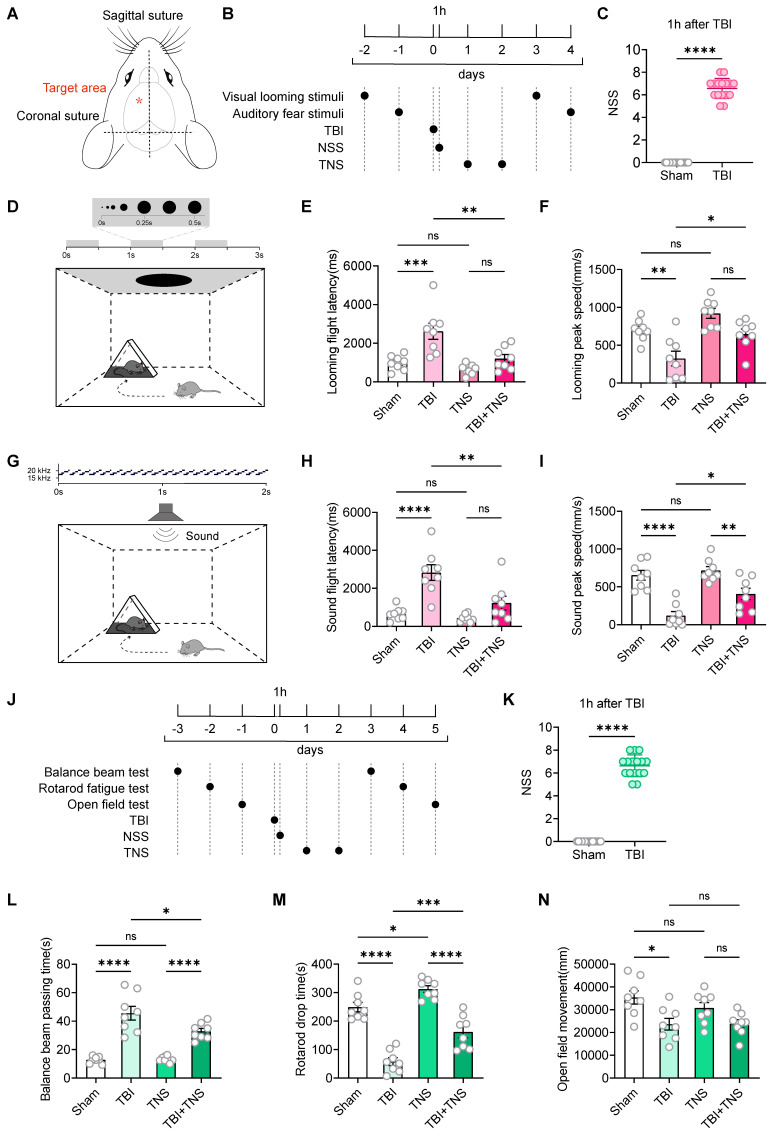
Effects of TNS on defensive arousal responses and motor abilities in TBI mice. (A) Schematic of the weight-drop modeling location. (B) Schematic diagram showing experimental design for defensive arousal response with TNS treatment after TBI. (C) One-hour post-surgery NSS for mice subjected to TBI modeling and for control mice not exposed to TBI (in defensive arousal response experiment) (*n* = 16 mice). (D) Schematic of the visual looming stimulus protocol, repeated over three cycles. (E and F) Latency (E) and peak escape velocity (F) following visual looming stimuli in the Sham group, TBI group, TNS group and TBI+TNS group (*n* = 8 mice). (G) Schematic of the auditory fear stimulus. (H and I) Latency (H) and peak escape velocity (I) following auditory fear stimuli in the Sham group, TBI group, TNS group and TBI+TNS group (*n* = 8 mice). (J) Schematic diagram showing experimental design for motor ability with TNS treatment after TBI. (K) One-hour post-surgery NSS for mice subjected to TBI modeling and for control mice not exposed to TBI (in motor ability experiment) (*n* = 16 mice). (L-N) Time to traverse the balance beam (L), drop time in the rotarod fatigue test (M) and total travel distance per unit time (10 minutes) in the open field test (N) within the Sham group, TBI group, TNS group, and TBI+TNS group (*n* = 8 mice). Data in (C, E, F, H, I, K-N) are expressed as means ± standard error of the mean (SEM). Statistical analyses were performed using one-way ANOVA (E, F, H, I, L-N). Statistical analyses were performed using Student *t*-tests (C, K) (****P < 0.0001; ***P < 0.001; ** P < 0.01; * *P* < 0.05; ns, not significant, *P* > 0.05). For *P*-values, see Table S4.

In this study, the innate defensive arousal response to audiovisual stimuli was used to assess defensive arousal in mice following TBI. The effectiveness of TNS in improving these responses post-TBI was investigated. The TBI model was established using a weight-drop method centered between the Bregma and Lambda points on the left side of the skull (Figure 1A). Following TBI induction, mice underwent TNS treatment at a frequency of 40 Hz, an intensity of 0.2 mA, and a pulse width of 200 μs for 60 minutes per day over two days, followed by visual and auditory stimuli (Figure 1B). The parameters and intensity of the TNS treatment were adapted from those employed in prior clinical research, with adjustments made to the electrical current strength to accommodate the tolerance levels of the mice 16. To assess the success of the model and the consistency of neurological impairments, the neurological severity score (NSS) was used one hour post-TBI to evaluate mice in both the Sham and TBI modeling groups (Figure 1C). Post-TNS, significant increases in neuronal activity were observed in certain brain regions, as indicated by elevated expression of the immediate early gene c-Fos (Figure S1).

The visual looming stimulus in a single experimental session consisted of a black disk that progressively enlarged over a three-second period, repeated over three cycles (Figure 1D). Experimental results demonstrated that TBI mice exhibited increased latency to escape and decreased escape speed in response to visual looming, indicative of reduced defensive responses. TNS treatment decreased escape latency and increased escape speed in response to visual looming stimuli in TBI mice, thereby restoring their defensive behavior. TNS also slightly enhanced the defensive response in normal mice, although the difference was not statistically significant compared to the Sham group (Figure 1E-F). The auditory stimuli in a single experimental session involved a sound of 70 dB that uniformly increased from 17 kHz to 20 kHz within 100 ms, played in a loop for 2 seconds (Figure 1G). Similar improvements were observed in auditory defensive responses after TNS treatment, analogous to those seen with visual looming stimuli (Figure 1H-I).

The behavioral paradigm for defensive arousal responses to visual and auditory stimuli encompasses two aspects: the sensitivity of mice to external sensory stimuli and their motor abilities. Following the creation of the TBI model, a noticeable impairment in motor functions was observed in the mice. To determine whether motor abilities influence the efficacy of TNS treatment on the defensive arousal responses in TBI mice, motor function tests were conducted. Commonly used behavioral paradigms to assess motor abilities include the balance beam test, the rotarod fatigue test, and the open field test. Prior to establishing the model, a screening of motor abilities was performed to select mice without statistical differences. After the TBI model was implemented, the mice received TNS treatment for two days, followed by tests of motor abilities (Figure 1J). The NSS was used to assess the efficacy of the modeling (Figure 1K). The results demonstrated that motor abilities were compromised in TBI mice, as shown by increased traversal time on the balance beam, decreased time before falling from the rotarod, and reduced total distance traveled in the open field test within a 10-minute period. TNS treatment led to improvements in motor functions in TBI mice, demonstrated by shortened balance beam traversal times and extended times on the rotarod. However, no significant statistical difference was observed in the total distance traveled in the open field test within 10 minutes (Figure 1L-N). Additionally, it was observed that TNS prolonged the rotarod latency in normal mice.

### TG Piezo2+ and Tac1+ neurons play a crucial role in restoring the defensive arousal responses of TBI mice treated with TNS

**Figure 2:**
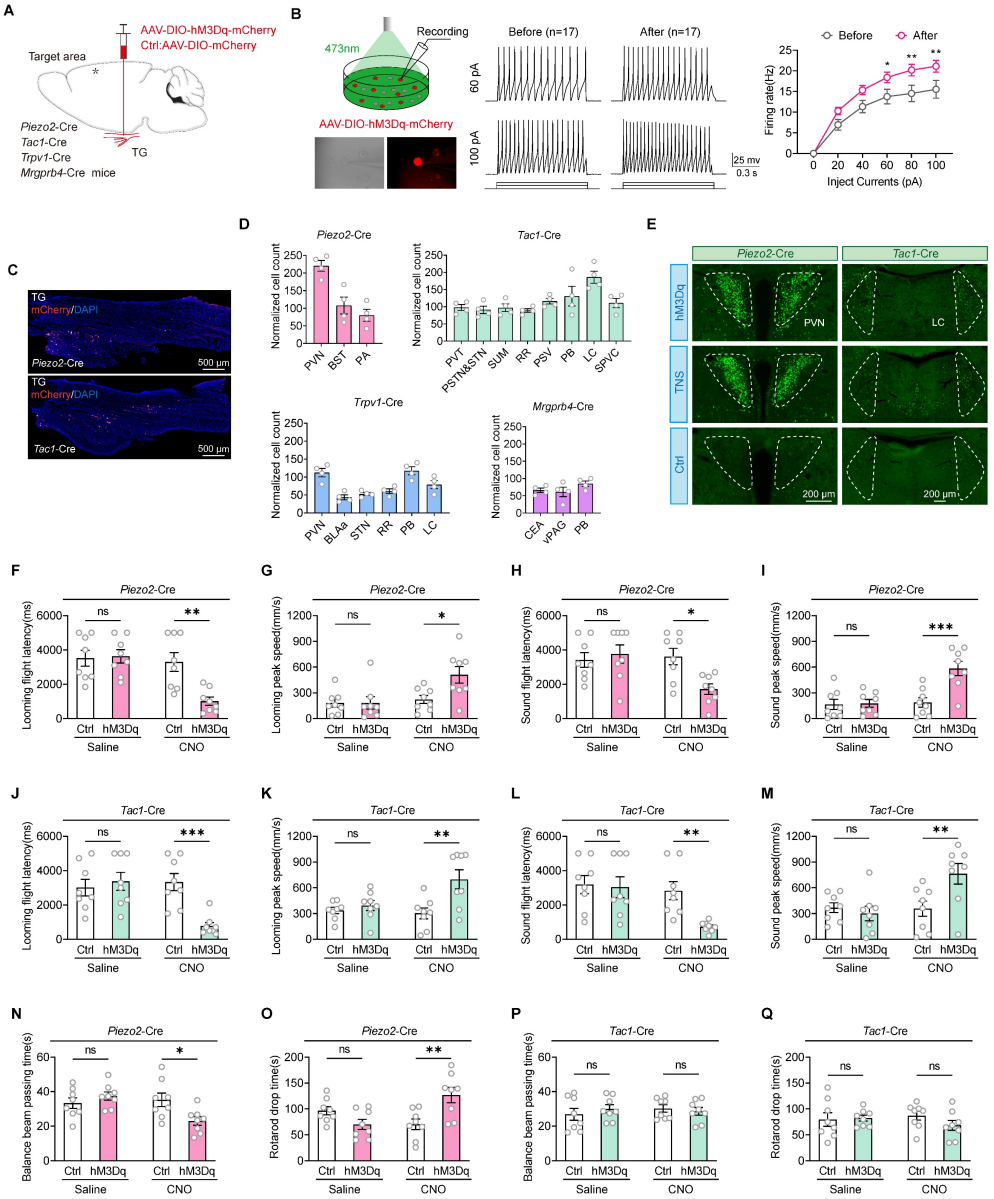
TG Piezo2+ and Tac1+ neurons are the key neurons in TNS treatment. (A) Schematic diagram depicting the injection of AAVs encoding Cre-dependent hM3Dq-mCherry into the TG of *Piezo2*-Cre, *Tac1*-Cre, *Trpv1*-Cre, and *Mrgprb4*-Cre mice. (B) An example train of action potentials recorded from TG neurons expressing hM3Dq-mCherry after perfusion of CNO (10 μM) in ACSF. (C) Schematic of longitudinal TG sections showing the expression of hM3Dq-mCherry in *Piezo2*-Cre (top) and *Tac1*-Cre (bottom) mice. (D) Total number of chemogenetically evoked c-Fos expression in various brain regions within 90 minutes in *Piezo2*-Cre (*n* = 4 mice), *Tac1*-Cre (*n* = 4 mice), *Trpv1*-Cre (*n* = 4 mice), and *Mrgprb4*-Cre (*n* = 4 mice) mice following injections of CNO. PVN, paraventricular hypothalamic nucleus; BST, bed nucleus of the stria terminalis; PA, posterior amygdalar nucleus; PVT, paraventricular thalamic nucleus; PSTN, parasubthalamic nucleus; STN, subthalamic nucleus; SUM, supramammillary nucleus; RR, retrorubral nucleus; PSV, principal sensory nucleus of the trigeminal; PB, parabrachial nucleus; LC, locus coeruleus; SPVC, spinal trigeminal nucleus, caudal part; BLAa, basolateral amygdalar nucleus, anterior part; CEA, central amygdaloid nucleus; vPAG, periaqueductal gray, ventral part. (E) Brain regions immunostained for a neuronal activation marker (c-Fos) 90 minutes after chemogenetic activation of Piezo2+ and Tac1+ TG neurons correspond to those regions activated by TNS. This experiment was repeated independently in 4 mice with similar results. (F and G) Latency (F) and peak escape velocity (G) of the visual looming defensive response in TBI mice with and without chemogenetic activation of Piezo2+ TG neurons (*n* = 8 mice). (H and I) Latency (H) and peak escape velocity (I) of the auditory stimulus defensive response in TBI mice with and without chemogenetic activation of Piezo2+ TG neurons (*n* = 8 mice). (J and K) Latency (J) and peak escape velocity (K) of the visual looming defensive response in TBI mice with and without chemogenetic activation of Tac1+ TG neurons (*n* = 8 mice). (L and M) Latency (L) and peak escape velocity (M) of the auditory stimulus defensive response in TBI mice with and without chemogenetic activation of Tac1+ TG neurons (*n* = 8 mice). (N and O) Time to traverse the balance beam (N) and drop time in the rotarod fatigue test (O) in TBI mice with and without chemogenetic activation of Piezo2+ TG neurons (*n* = 8 mice). (P and Q) Time to traverse the balance beam (P) and drop time in the rotarod fatigue test (Q) in TBI mice with and without chemogenetic activation of Tac1+ TG neurons (*n* = 8 mice). Data in (B, D, F-Q) are expressed as means ± SEM. The broken white lines in the section images represent boundaries of brain regions. Statistical analysis in (B) was performed using two-way ANOVA (**P < 0.01; *P < 0.05). Statistical analyses in (F-Q) were performed using Student *t*-tests (***P < 0.001; **P < 0.01; *P < 0.05; ns, not significant, *P* > 0.05). For *P*-values, see Table S4.

To further investigate whether improvements in motor abilities are essential for the rehabilitation of defensive arousal responses observed in TBI mice treated with TNS, and to determine if TNS can independently revitalize the sensitivity of mice to external sensory stimuli (which requires unrestricted motor abilities for behavioral experiments), the focus was on identifying key neurons in the TG that could improve defensive arousal responses without altering motor abilities. Attention was particularly given to the looming visual stimuli paradigm. Utilizing data from published single-cell sequencing repositories, neuron types within the TG were identified based on their distinct sensory functions, including mechanical sensation (*Piezo2*-Cre), peptidergic nociception (*Tac1*-Cre), thermal pain perception (*Trpv1*-Cre), and itch response (*Mrgprb4*-Cre) 20, 21. Bilateral injections of adeno-associated virus (AAV) vectors AAV2/9-hSyn-DIO-hM3Dq-mCherry were administered into the TG of these Cre line mice. The control group received injections of AAV2/9-hSyn-DIO-mCherry (Figure 2A). The efficacy of clozapine N-oxide (CNO) for chemogenetic activation of action potential firing was subsequently confirmed in acute brain slices (Figure 2B). The mCherry labeling was confirmed by immunostaining of TG sections (Figure 2C). Following chemogenetic activation, the four neuronal subtypes exhibited significantly increased activity in various brain regions, as indicated by the upregulation of c-Fos expression (Figure 2D; Figure S2).

Compared to mice injected with the control virus, neuronal activity in the PVN of *Piezo2*-Cre mice and in the LC of *Tac1*-Cre mice was elevated after intraperitoneal injection of CNO at a dosage of 1 mg/kg. This elevation correlates with regions showing significant increases in neuronal activity following TNS (Figure 2E). Prior to virus injection, sensory stimulus screening was conducted, followed by TBI modeling and intraperitoneal injection of CNO two weeks later (Figure S3A). Compared to mice injected with the control virus, *Piezo2*-Cre mice in the hM3Dq group showed a reduction in escape latency and an increase in escape speed during defensive arousal responses to visual and auditory stimuli (Figure 2F-I), and *Tac1*-Cre mice exhibited similar improvements (Figure 2J-M). However, no improvement in defensive arousal response was observed in *Trpv1*-Cre (Figure S3B-E) and *Mrgprb4*-Cre (Figure S3F-I) mice. Given the correlation between activated nuclei and behavioral outcomes with TNS, it is hypothesized that Piezo2+ and Tac1+ neurons play significant roles in the effects of TNS.

Using the same strategy, the study also tested whether motor abilities improved for *Piezo2*-Cre and *Tac1*-Cre mice (Figure S3J). In the *Piezo2*-Cre hM3Dq group, compared to the control group, the time taken to traverse the balance beam was reduced, and the time to fall off the rotarod was extended (Figure 2N-O), indicating improved motor functions in TBI mice. However, in the *Tac1*-Cre hM3Dq group, compared to the control group, there were no significant statistical differences in the time taken to traverse the balance beam or the time to fall off the rotarod (Figure 2P-Q), showing no improvement in motor abilities of TBI mice. Therefore, it is proposed that the activation of Piezo2+TG revives the defensive arousal responses in TBI mice by augmenting their motor abilities, whereas the Tac1+TG renews the defensive arousal response in TBI mice by increasing their sensitivity to external sensory stimuli.

### The Tac1+TG-LC-SC pathway repairs the response of TBI mice to defensive arousal induced by visual looming stimuli

**Figure 3:**
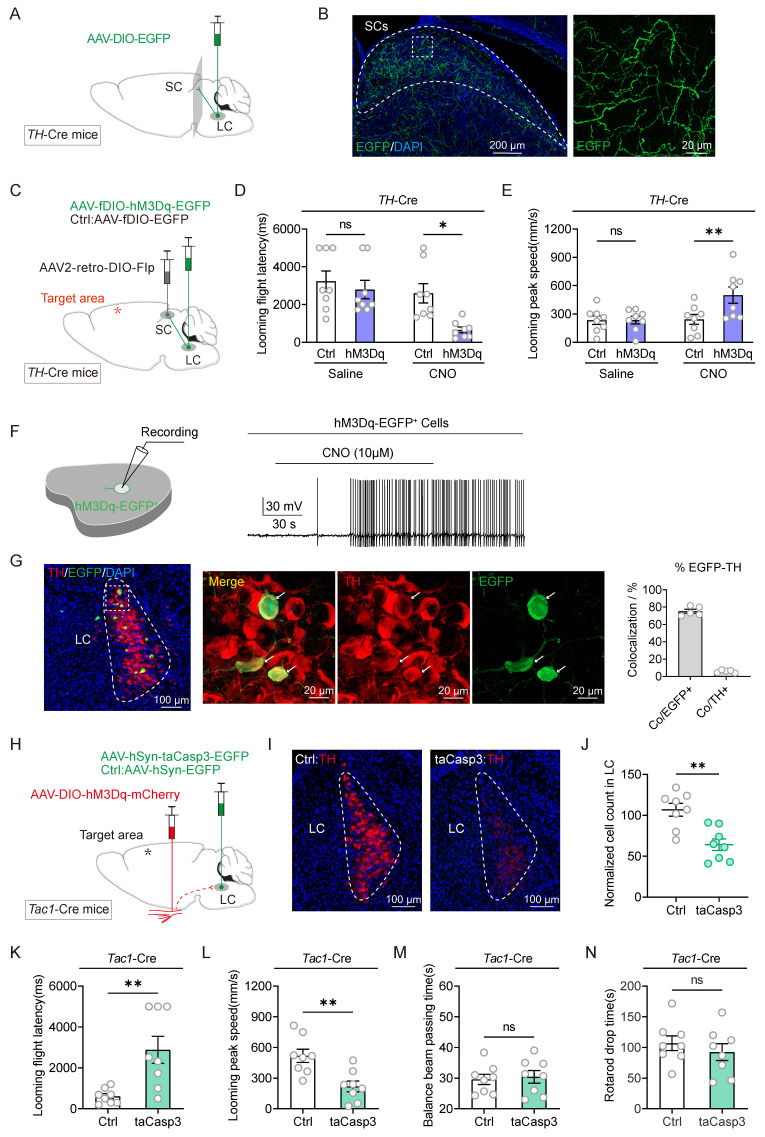
Tracing and modulation of the Tac1:TG-LC-SC pathway. (A) Schematic diagram showing injection of AAV2/9-DIO-EGFP into the LC of *TH*-Cre mice. (B) Example coronal sections (left) and magnified field (right) showing EGFP+ axonal projections of TH+ LC neurons in the ipsilateral SC. (C) Viral injection strategy to express hM3Dq-mCherry in SC-projecting TH+ LC neurons in *TH*-Cre mice. (D and E) Latency (D) and peak escape velocity (E) of the visual looming defensive response in TBI mice without and with chemogenetic activation of SC-projecting TH+ LC neurons (*n* = 8 mice). (F) Example trace of action potential firing demonstrates the effectiveness of CNO in activating hM3Dq-expressing LC neurons in acute brain slices. (G) Example coronal brain section (left) and the magnified fields (middle) showing the distribution of SC-projecting (EGFP+) TH+ neurons in the LC. Specificity (Co/EGFP+) and efficiency (Co/TH+) for hM3Dq-EGFP to label TH+ LC neurons (right). (H) Viral injection strategy to express hM3Dq-mCherry in Tac1+ TG neurons in *Tac1*-Cre mice, with and without taCasp3 ablation of LC neurons. (I) Example coronal brain section showing TH+ LC neurons with (right) and without (left) taCasp3 ablation of LC neurons. (J) Quantitative analysis of TH+ LC neurons with and without taCasp3 ablation of LC neurons (*n* = 8 mice). (K and L) Latency (K) and peak escape velocity (L) of the visual looming defensive response in TBI mice with and without taCasp3 ablation of LC neurons (*n* = 8 mice). (M and N) Time to traverse the balance beam (M) and rotarod fatigue test fall time (N) in TBI mice with and without taCasp3 ablation of LC neurons (*n* = 8 mice). Data in (D, E, G, J-N) are expressed as means ± SEM. The broken white lines in the section images represent boundaries of brain regions. Statistical analyses were performed using Student *t*-tests (D, E, J-N) (**P < 0.01; *P < 0.05; ns, not significant, *P* > 0.05). For *P*-values, see Table S4.

Chemical activation of Tac1+TG neurons led to increased expression of c-Fos in the LC (Figure 2E). Previous work from our group demonstrated that following the injection of the retrograde trans-synaptic virus HSV-EGFP into the TG, infected cell bodies were identified in the LC 19, suggesting the LC as a downstream projection target of Tac1+TG, thereby confirming the existence of the Tac1+TG-LC pathway. The SC serves as a critical hub for processing visual threats and initiating innate defensive behaviors. Optogenetic activation of neurons in the SC can rapidly trigger freezing and escape behaviors 10. Consequently, the role of the SC in mediating the defensive arousal response to visual looming stimuli in TBI mice through Tac1+TG neurons is under investigation. The LC consists of noradrenergic neurons, identifiable by tyrosine hydroxylase (TH) staining. In *TH*-Cre mice, AAV2/9-hSyn-DIO-EGFP was injected unilaterally into the LC (Figure 3A). Consistent with previous research 22, TH+ LC neurons were observed to project diffusely to the SC (Figure 3B). Therefore, it is speculated that the SC is involved in the neural circuit activated by Tac1+TG neurons.

To determine whether the Tac1+TG-LC-SC pathway contributes to the defensive arousal induced by visual looming stimuli in TBI mice, chemogenetic activation and ablation strategies were employed. In the experimental group, *TH*-Cre mice received bilateral LC injections of AAV2/9-fDIO-hM3Dq-EGFP, while the control group received AAV2/9-fDIO-EGFP injections. Both groups also received bilateral injections of AAV2-retro-DIO-Flp into the SC, ensuring the selective expression of hM3Dq-EGFP in TH+ LC neurons with projections to the SC (Figure 3C). Two weeks after viral injection, TBI modeling was performed, including mice with NSS ratings between 6 and 8. Activating these SC-projecting TH+ LC neurons significantly shortened the escape latency and increased the peak escape speed of TBI mice in response to visual looming stimuli (Figure 3D-E), demonstrating that activation of these neurons repairs the visual stimulus defensive arousal response in TBI mice. Chemogenetic activation of neuronal firing by CNO (10 μM) was confirmed using slice physiology (Figure 3F). To examine the specificity and efficiency of hM3Dq-EGFP labeling, EGFP and TH were stained in the LC of *TH*-Cre mice (Figure 3G). A large proportion of EGFP+ cells (75.3% ± 2.4%, 5 mice) were positive for TH, representing a minor subset of TH+ LC neurons.

The next experiment was conducted to evaluate whether inactivating the LC through apoptosis-inducing viral-mediated localized lesioning could reduce the defensive responses triggered by Tac1+TG activation. In *Tac1*-Cre mice, either AAV2/9-hSyn-taCasp3-TEVp-EGFP or AAV2/9-hSyn-EGFP was injected into the bilateral LC, while Cre-dependent AAV2/9-hSyn-DIO-hM3Dq-mCherry was injected into the bilateral TG (Figure 3H). The efficiency of bilateral TH+ LC neuron ablation was quantified by counting TH+ neurons (Figure 3I-J). The results indicated that partial ablation of the LC attenuated the defensive responses induced by Tac1+TG activation, evidenced by extended escape latency and reduced maximum escape speeds compared to the control group (Figure 3K-L). This combinatorial strategy had no significant impact on the motor abilities of TBI mice (Figure 3M-N). These findings, combined with the results of viral tracing and manipulation of the Tac1+TG-LC-SC pathway, structurally and functionally demonstrated that Tac1+TG improves the visual looming-induced defensive arousal response in TBI mice via this pathway.

### TNS rebuilds the sensitivity of the SC receptive field through the Tac1+TG-LC-SC pathway

The SC receives direct inputs from the retina and is essential for detecting salient visual information, playing a pivotal role in the perception of visual looming stimuli. The responsiveness of SC neurons is crucial for determining how animals react behaviorally to visual stimuli, and this responsiveness is profoundly influenced by the animal's internal state and level of arousal. Given that the Tac1+TG-LC-SC pathway does not affect motor ability, it was previously hypothesized that this pathway might restore the defensive arousal responses in TBI mice by increasing their sensitivity to external sensory stimuli. To assess the sensitivity of TBI mice to external visual stimuli without the influence of motor abilities, a behavioral paradigm based on visual stimulus sensitivity curves was developed to evaluate the sensitivity of the SC receptive field.

**Figure 4:**
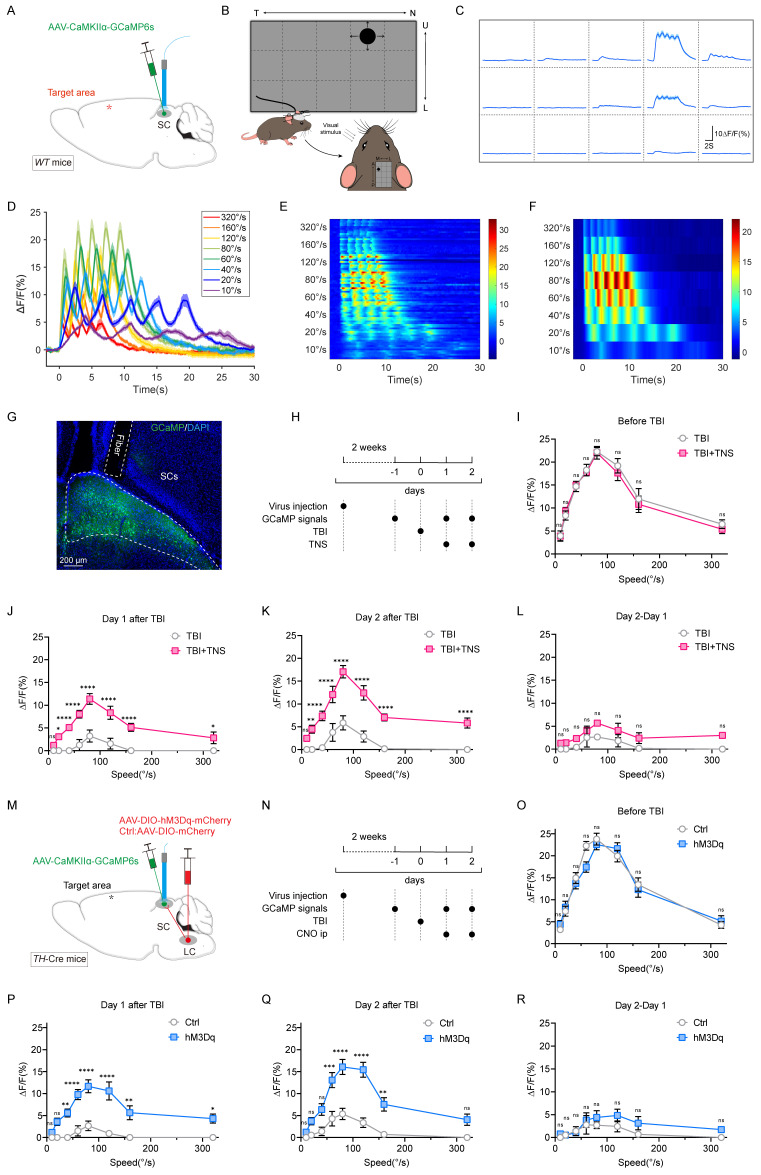
TNS and chemogenetic activation of TH+ LC neurons restore receptive field sensitivity in the SC of TBI mice. (A) Schematic of AAV2/9-CaMKIIα-GCaMP6s injection and optical fiber implantation in the SC of *WT* mice. (B) Schematic of the behavioral paradigm for visual stimuli at varying rates. (C) After fixing the screen and mouse position, the screen shows varying GCaMP signals intensities at different positions; the schematic indicates that position 4 is the most sensitive receptive field in the SC. (D) GCaMP signals waveforms in response to visual stimuli at various rates (320 °/s, 160 °/s, 120 °/s, 80 °/s, 60 °/s, 40 °/s, 20 °/s, 10 °/s), thick lines indicate mean of 10 trials. (E) Heat map showing 10 trials of normalized GCaMP fluorescence changes (ΔF/F) aligned with visual stimuli for different rates (320 °/s, 160 °/s, 120 °/s, 80 °/s, 60 °/s, 40 °/s, 20 °/s, 10 °/s) (*n* = 4 mice). (F) Heat map showing average GCaMP fluorescence changes (ΔF/F) of different rates aligned with visual stimuli (*n* = 4 mice). (G) Optical fiber track above GCaMP6s-expressing neurons in SC of *WT* mice. (H) Schematic showing experimental design for GCaMP signals response to visual stimuli at various rates in TBI mice with or without TNS treatment. (I-K) GCaMP signals respond to visual stimuli at different rates, measured respectively before TBI (I), one day after TBI (J), and two days after TBI (K) with and without TNS treatment (*n* = 4 mice). (L) Evaluation of the effect of the second TNS application by subtracting the GCaMP signals on day 1 from day 2 post-TBI (*n* = 4 mice). (M) Schematic diagram showing position of virus injection of CaMKIIα-GCaMP6s, optical fiber implantation in the SC and hM3Dq-mCherry in unilateral TH+ LC neurons. (N) Schematic showing experimental design for GCaMP signals response to visual stimuli at various rates in TBI mice with or without CNO activation. (O-Q) GCaMP signals respond to visual stimuli at different rates, measured respectively before TBI (O), one day after TBI (P), and two days after TBI (Q) with and without chemogenetic activation of TH+ LC neurons (*n* = 4 mice). (R) Evaluation of the effect of the second chemogenetic activation of TH+ LC neurons application by subtracting the GCaMP signals on day 1 from day 2 post-TBI (*n* = 4 mice). Data in (I-L, O-R) are expressed as means ± SEM. Statistical analysis in (I-L, O-R) were performed using two-way ANOVA (****P < 0.0001; ***P < 0.001; **P < 0.01; *P < 0.05; ns, not significant, *P* > 0.05). For *P*-values, see Table S4.

To monitor SC neuronal activity during visual stimuli, AAV2/9-CaMKIIα-GCaMP6s was injected into the SC of wild-type (*WT*) mice, and a fiber optic was implanted to perform fiber photometry for recording GCaMP signals (Figure 4A). By determining the most sensitive receptive field location for mouse vision (Figure 4B-C), looming stimuli at varying rates were applied to this location. The GCaMP signal waveforms for each looming speed were represented by superimposed data from five repeated stimuli (Figure 4D-F). Data selection was based on histological results that confirmed correct virus injection locations and appropriate fiber placements (Figure 4G).

Prior to TBI modeling, mice exhibited a stable bell-shaped response curve to various rates of visual looming stimuli, with the peak response occurring at a looming rate of 80 °/S. In the TBI group, this response curve diminished or disappeared, showing slight improvement over time (one or two days later). After TNS treatment, the curve gradually recovered, indicating a faster restoration of sensitivity to visual stimuli (Figure 4H-K). The effect of TNS on the second day was assessed by the GCaMP signal difference between day 2 and day 1. While the TBI+TNS group showed a slightly higher signal compared to the TBI group, the difference was not statistically significant (Figure 4L). These experimental results revealed a decline in the defensive arousal response of the SC to innate visual stimuli in mice with impaired arousal levels post-TBI. However, TNS treatment improved this response, suggesting that TNS repairs the sensitivity of the SC receptive fields in TBI mice, thereby restoring their defensive arousal response to visual stimuli.

Next, to verify whether TNS restores the sensitivity of the SC receptive field in TBI mice via the TG-LC-SC pathway, AAV2/9-hSyn-DIO-hM3Dq-mCherry was injected unilaterally into the LC of *TH*-Cre mice, and AAV2/9-CaMKIIɑ-GCaMP6s was injected into the SC on the same side, followed by fiber implantation (Figure 4M). Continuous intraperitoneal injections of CNO over two days were administered to activate LC neurons. GCaMP signal curves in response to varying rates of visual stimuli gradually recovered over time in the hM3Dq group with chemogenetic activation, approaching pre-modeling baseline levels. In contrast, response curves in the control group diminished or disappeared post-TBI, showing only slight improvement over time (Figure 4N-Q). The second chemogenetic activation of the LC further improved the visual response curve, though the difference was not statistically significant (Figure 4R). These findings suggest that activated TH+ LC neurons repair the sensitivity of the SC receptive field in TBI mice. When combined with results from previous behavioral experiments, which demonstrated that Tac1+TG activation improved defensive responses but did not restore motor abilities in TBI mice, it is likely that TNS reinstates the defensive arousal response to visual stimuli in TBI mice by improving SC receptive field sensitivity via the Tac1+TG-LC-SC pathway.

### TNS upregulates noradrenaline content in the SC via the LC-SC pathway

The LC serves as a primary source of noradrenergic innervation within the cortex 23, and research has shown that the LC-SC pathway modulates stress-induced effects on innate fear-related defensive behaviors by regulating noradrenaline (NE) receptors in the downstream SC 22. To monitor the release of NE, a genetically encoded GPCR-activated NE sensor (NE2h) was employed to detect the dynamics of NE in the SC following activation of the LC-SC pathway 24.

**Figure 5:**
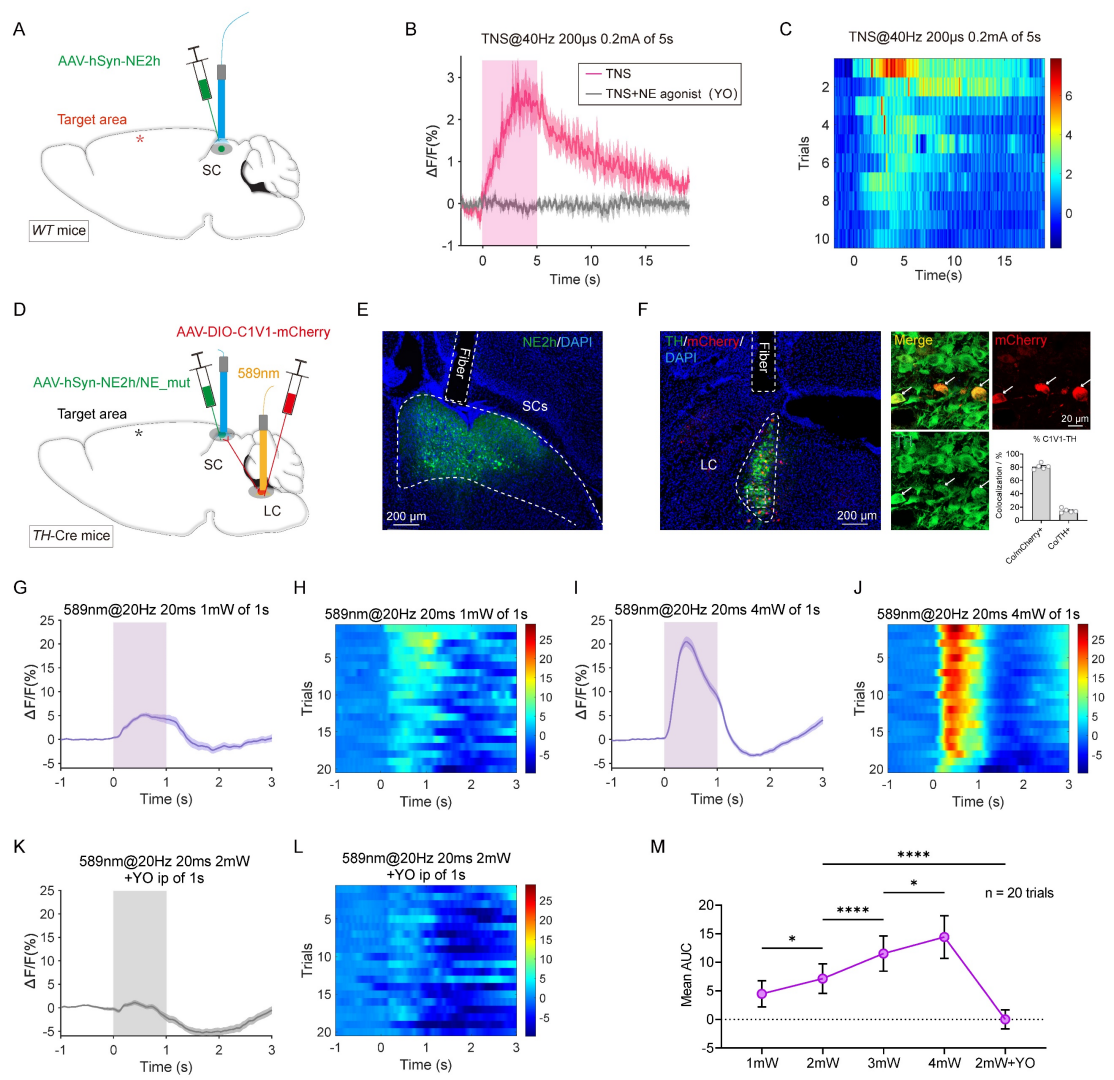
TNS upregulates the NE level in SC of TBI mice by activating LC-SC pathway. (A) Schematic of AAV2/9-hSyn-NE2h injection and optical fiber implantation in the SC of *WT* mice. (B) NE fluorescence changes (ΔF/F) in the SC during TNS and after intraperitoneal injection of the NE receptor antagonist yohimbine (YO, 2 mg/kg) (*n* = 5 mice). (C) Heat map showing 10 trials of normalized NE fluorescence changes aligned by TNS treatment (*n* = 5 mice). (D) Schematic of the injection of AAV2/9-hSyn-NE2h/NE_mut into the SC and AAV2/9-DIO-C1V1-mCherry into the TH+ LC of *TH*-Cre mice, followed by optical fiber implantation above the SC for fiber photometry recording and optical fiber implantation above the TH+ LC neurons for optogenetic activation. (E) Example coronal section with an optical-fiber track above the SC neurons expressing NE sensor. (F) Example coronal brain section (left) and the magnified fields (right) showing optical fiber track and the expression of C1V1-mCherry in TH+ LC neurons of *TH*-Cre mice. Specificity (Co/mCherry+) and efficiency (Co/TH+) for C1V1-mCherry to label TH+ LC neurons (right bottom). (G and H) NE signals (ΔF/F) (G) and heat map (H) of 20 trials evoked by photostimulation of TH+ LC neurons with 1 mW laser power (589 nm, 20 ms, 20 Hz, 1 pulse of 1 second) (*n* = 5 mice). (I and J) NE signals (ΔF/F) (I) and heat map (J) of 20 trials evoked by photostimulation of TH+ LC neurons with 4 mW laser power (*n* = 5 mice). (K and L) NE signals (ΔF/F) (K) and heat map (L) of 20 trials evoked by photostimulation of TH+ LC neurons using 2 mW laser power, 15 minutes after intraperitoneal injection of the NE receptor antagonist YO (2 mg/kg) (*n* = 5 mice). (M) Statistical graph showing the area under the curve (AUC) of NE signals within 1 second under different photostimulation powers and with the use of an antagonist (*n* = 20 trials). Data in (M) is expressed as means ± SEM. The broken white lines in the section images represent boundaries of brain regions. Statistical analyses were performed using one-way ANOVA (M) (****P < 0.0001; *P < 0.05). For *P*-values, see Table S4.

In *WT* mice, AAV2/9-hSyn-NE2h was injected unilaterally into the SC, followed by fiber implantation (Figure 5A). Two weeks later, TBI modeling was performed. When TBI mice were subjected to continuous TNS (0.2 mA, 40 Hz) for 5 seconds, the fluorescence signal from the NE sensor in the SC was regenerated (Figure 5B-C). After the stimulation ceased, NE levels gradually returned to baseline within approximately 10 seconds, indicating that TNS has a delayed effect. Intraperitoneal injection of the NE receptor antagonist yohimbine (YO, 2 mg/kg) abolished the NE signal induced by TNS, and no significant change was observed in the NE signal in the SC when the forelimbs of the mouse were subjected to electrical stimulation with the same parameters (Figure S4A-B).

To explore whether activation of the LC-SC pathway triggers the release of NE in the SC, AAV2/9-DIO-C1V1-mCherry and AAV2/9-hSyn-NE2h were injected into the LC and SC of *TH*-Cre mice, respectively. Optical fibers were then implanted above both the LC and SC (Figure 5D). Immunohistochemistry was performed to verify the expression of the viruses and the placement of the fibers (Figure 5E). The specificity and efficiency of C1V1-mCherry labeling were evaluated through mCherry and TH staining in the LC (Figure 5F), revealing that a substantial percentage of mCherry-positive cells (80.7% ± 1.9%, 5 mice) were TH-positive.

In TBI mice, stimulation of TH+ LC neurons with single light pulses (589 nm, 20 ms, 20 Hz, 1-4 mW) temporarily increased the fluorescence of the NE sensor in the SC. The intensity of the fluorescence signal correlated positively with the intensity of the light stimulation (Figure 5G-J, M; Figure S4C-F). As a control, no significant fluorescence changes were observed in SC neurons expressing a mutated NE sensor (NE_mut), which lacks sensitivity to noradrenaline (Figure S4G-H). Additionally, intraperitoneal injection of the NE receptor antagonist YO abolished the NE signal induced by light stimulation (Figure 5K-M). These findings confirm that activation of the LC-SC pathway specifically triggers the release of NE in the SC. This indicates that TNS effectively rebuilds the NE content in the SC via the LC-SC pathway, thereby improving the defensive arousal response to visual stimuli in TBI mice.

### The Piezo2+TG-PVN-SNc-DS pathway restores motor abilities and visual defensive arousal responses in TBI mice

Chemogenetic activation of Piezo2+TG neurons resulted in a notable increase in neuronal activity within the PVN, a response also observed during TNS treatment. This indicates that TNS likely engages the Piezo2+TG-PVN pathway. Previous work from our group confirmed the existence of the TG-PVN-SNc pathway by identifying infected cell bodies in the PVN following injection of the retrograde trans-synaptic virus HSV-EGFP into the TG 19. The nigrostriatal pathway, a key component of the midbrain dopaminergic system, originates in the SNc and projects to the DS, specifically the caudate and putamen. This pathway plays a pivotal role in controlling voluntary movement and facilitating motor skills learning 25, 26. Given that chemogenetic activation of Piezo2+TG neurons improves motor abilities in TBI mice, further exploration is warranted to determine whether TNS restores motor function and defensive arousal responses in TBI mice through the Piezo2+TG-PVN-SNc-DS neural circuit.

**Figure 6:**
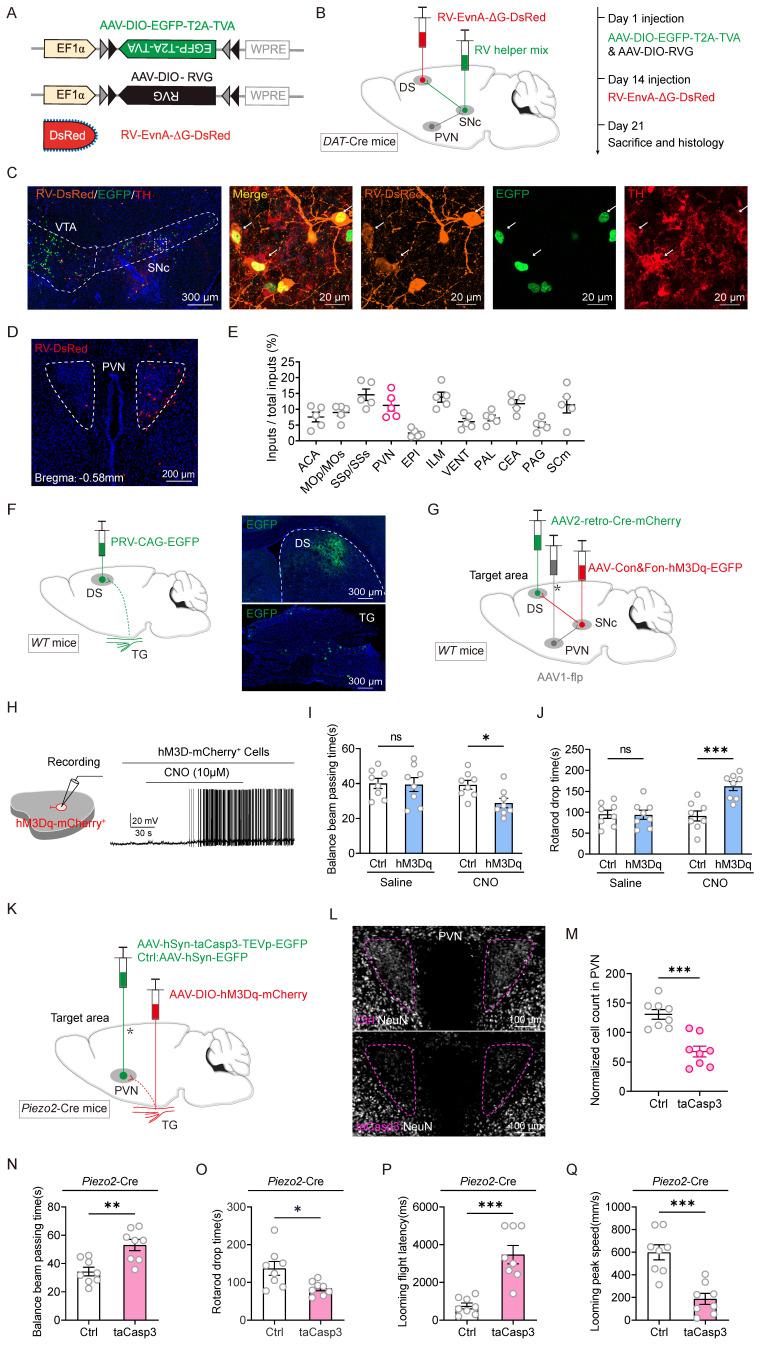
Tracing and modulation of the Piezo2:TG-PVN-SNc-DS pathway. (A) Schematic diagram showing viral injection strategy for retrograde tracing of DS neurons. (B) Schematic diagrams showing the AAV helpers and RV used for injection (left) and the schedule of AAV/RV injections (right). (C) Example coronal brain section and magnified field showing the triply labeled relay cells in the SNc of *DAT*-Cre mice. (D) Example coronal brain section showing DsRed-labeled neurons in the PVN. (E) Fractions of total RV-labeled cells in different brain regions projecting monosynaptically to the SNc-DS, normalized by dividing by the total number of DsRed+ cells in these brain regions (*n* = 5 mice). ACA, anterior cingulate area; MOp/Mos, primary motor area/secondary motor area; SSp/SSs, primary somatosensory area/supplemental somatosensory area; EPI, epithalamus; ILM, intralaminar nuclei of the dorsal thalamus; VENT, ventral group of the dorsal thalamus; PAL, pallidum; CEA, central amygdaloid nucleus; PAG, periaqueductal gray; SCm, superior colliculus, motor related. (F) Schematic diagram showing injection of PRV-CAG-EGFP in DS (left). Example coronal brain section showing EGFP-labeled starter cells in the DS (right top) and the example longitudinal TG sections showing the distribution of retrogradely PRV-labeled neurons in TG (right bottom). (G) Schematic diagram showing injection of AAV2/9-Con&Fon-hM3Dq into SNc, AAV1-Flp into PVN and AAV2-retro-Cre into DS of *WT* mice for pathway activation. (H) An example train of action potentials recorded from SNc neurons expressing hM3Dq-mCherry after perfusion of CNO (10 μM) in ACSF. (I and J) Time to traverse the balance beam (I) and drop time in the rotarod fatigue test (J) in TBI mice with and without chemogenetic activation of PVN-SNc-DS pathway (*n* = 8 mice). (K) Schematic diagram showing injection of AAV2/9-DIO-hM3Dq-mCherry in Piezo2+ TG neurons in *Piezo2*-Cre mice, with and without taCasp3 ablation of PVN neurons. (L) Example coronal brain section showing PVN neurons without (top) and with (bottom) taCasp3 ablation of PVN neurons. (M) Quantitative analysis of PVN neurons with and without taCasp3-mediated apoptosis (*n* = 8 mice). (N and O) Time to traverse the balance beam (N) and rotarod fatigue test fall time (O) in TBI mice with and without taCasp3 ablation of PVN neurons (*n* = 8 mice). (P and Q) Latency (P) and peak escape velocity (Q) of the visual looming defensive response in TBI mice with and without taCasp3 ablation of PVN neurons (*n* = 8 mice). Data in (E, I, J, M-Q) are expressed as means ± SEM. The broken lines in the section images represent boundaries of brain regions. Statistical analyses were performed using Student *t*-tests (I, J, M-Q) (***P < 0.001; **P < 0.01; *P < 0.05). For *P*-values, see Table S4.

A recombinant rabies virus (RV) vector was employed for monosynaptic retrograde tracing to identify neurons in the DS projecting back to the PVN and to verify whether DAT+ neurons in the SNc serve as an intermediate link between PVN and DS neurons. Cre-dependent helper viruses (AAV2/9-Ef1ɑ-DIO-TVA-EGFP and AAV2/9-Ef1ɑ-DIO-RVG) were injected into the SNc of *DAT*-Cre mice. Fourteen days later, EnvA-RV-ΔG-DsRed was injected into the DS (Figure 6A-C). Retrogradely labeled DsRed neurons were observed in PVN sections (Figure 6D). These retro-labeled neurons were predominantly located in the PVN, somatosensory area (SS), and intralaminar nuclei of the dorsal thalamus (ILM) (Figure 6E). To further determine whether the TG is involved in the PVN-SNc-DS pathway, pseudorabies virus (PRV) retrograde tracing was conducted by injecting PRV-CAG-EGFP into the DS. Retro-labeled EGFP+ neurons from the DS were observed in the TG (Figure 6F). These viral tracing results indicate that the tri-synaptic PVN-SNc-DS pathway plays a role in mediating the improvements in motor abilities observed in TBI mice following TNS treatment.

To validate this hypothesis that the PVN-SNc-DS pathway mediates motor recovery in TBI mice, a pathway-specific chemogenetic activation strategy was employed. In *WT* mice, AAV2-retro-Cre was injected into the DS, AAV1-Flp into the PVN, and either AAV2/9-DIO-Con&Fon-hM3Dq-mCherry or AAV2/9-DIO-Con&Fon-mCherry into the SNc (Figure 6G). This configuration allowed for the expression of hM3Dq-mCherry in the SNc only when there were both anterograde projections from the PVN and retrograde projections from the DS. Activation of neuronal firing by CNO (10 μM) was confirmed through slice physiology (Figure 6H). Chemogenetic activation of this pathway significantly improved motor abilities in TBI mice, as evidenced by a reduction in the time required to traverse the balance beam and an increase in endurance on the rotarod (Figure 6I-J). These results suggest that stimulating the PVN-SNc-DS pathway restores motor capabilities in TBI mice, further supporting the involvement of this neural circuit in mediating the therapeutic effects of TNS on motor function recovery.

To evaluate the role of the Piezo2+TG-PVN-SNc-DS pathway in the restoration of motor abilities in TBI mice following TNS treatment, apoptosis-inducing viruses were employed to selectively ablate the PVN and determine whether this would diminish motor improvements induced by Piezo2+TG activation. AAV2/9-hSyn-DIO-hM3Dq-mCherry was injected bilaterally into the TG of *Piezo2*-Cre mice, while either AAV2/9-hSyn-taCasp3-TEVp-EGFP (for apoptosis induction) or AAV2/9-hSyn-EGFP (control) was injected bilaterally into the PVN (Figure 6K). The efficiency of bilateral PVN neuron ablation was assessed by counting the number of neurons stained with a neuronal marker (Figure 6L-M).

The experiment demonstrated that partial ablation of the PVN significantly curtailed the motor skill rehabilitation triggered by Piezo2+TG activation, as evidenced by longer balance beam traversal times and shorter durations on the rotarod compared to the control group (Figure 6N-O). This ablation also weakened the restoration of defensive responses facilitated by Piezo2+TG activation (Figure 6P-Q). When the PVN was partially deactivated, stimulation of Piezo2+TG neurons failed to restore motor abilities or defensive arousal responses to visual stimuli in TBI mice. These observations indicate that TNS reinstates motor functions and defensive arousal responses to visual stimuli in TBI mice primarily through the Piezo2+TG-PVN-SNc-DS pathway.

### TNS triggers dopamine release in the DS via the PVN-SNc-DS pathway

**Figure 7:**
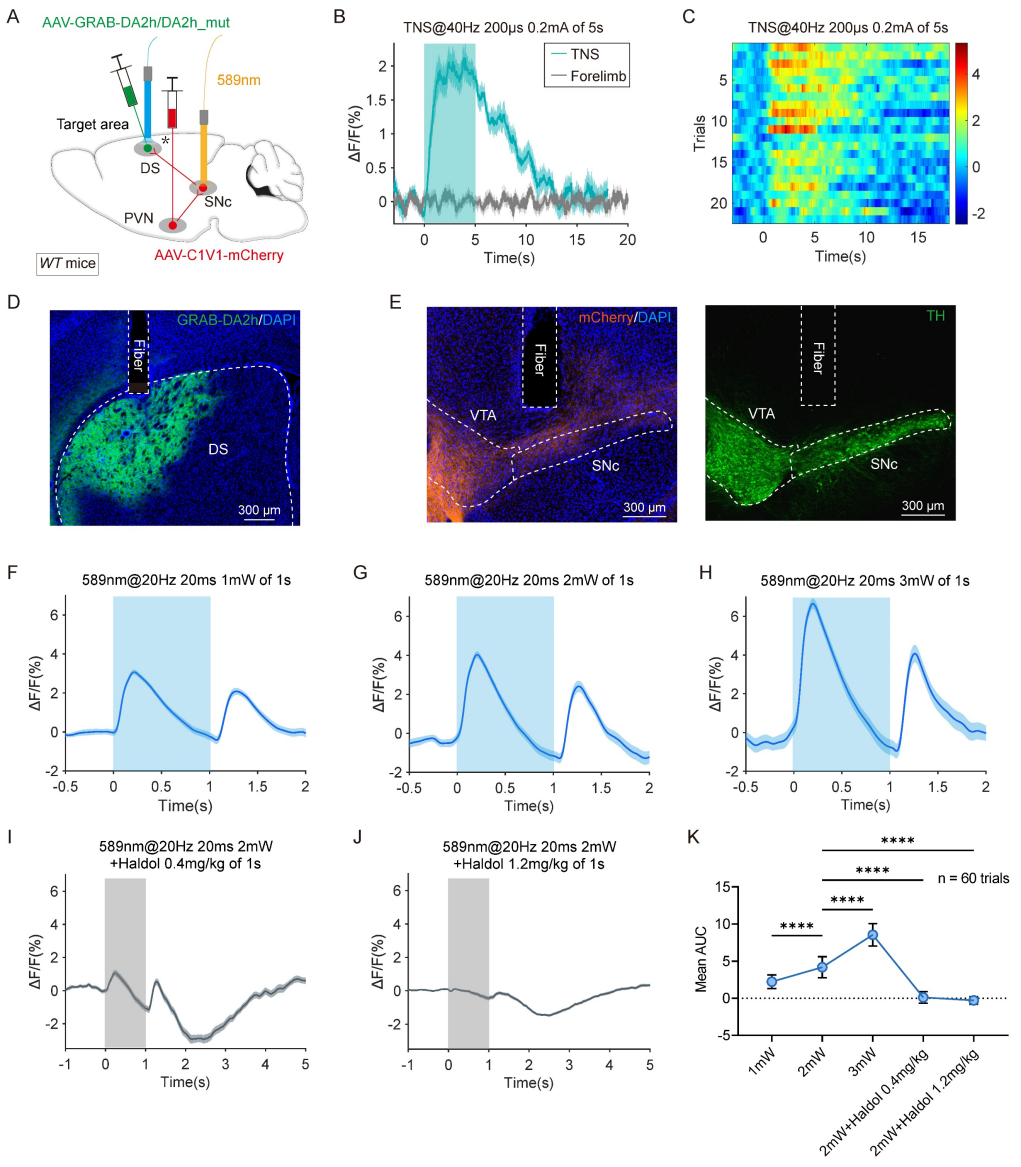
TNS upregulates the DS level in DA of TBI mice by activating PVN-SNc-DS pathway. (A) Schematic diagram showing injections of AAV2/9-C1V1-mCherry in the PVN and AAV2/9-GRAB-DA2h/DA2h_mut in the DS of *WT* mice. followed by optical fiber implantation above the SNc and DS to apply optogenetic activation and fiber photometry recording, respectively. (B) GRAB-DA2h signals (ΔF/F) of 20 trials in the DS during TNS and electrical stimulation of forelimbs (Stimulation parameters are consistent with TNS) of TBI mice. (C) Heat map showing 20 trials of normalized GRAB-DA2h signals aligned with TNS treatment. (D) Example coronal section with an optical-fiber track above the DS neurons expressing GRAB-DA2h sensor. This experiment was repeated independently in 6 mice with similar results. (E) Example coronal section of ventral midbrain showing an optical-fiber track above the C1V1-mCherry+ axon terminals in the SNc (left), the boundary of which was determined according to the immunofluorescence of TH (right). This experiment was repeated independently in 6 mice with similar results. (F-H) GRAB-DA2h signals (ΔF/F) (60 trials) evoked by photostimulation of the PVN-SNc pathway with 1 mW (F), 2 mW (G), 3 mW (H) laser power (589 nm, 20 ms, 20 Hz, 1 pulse of 1 second) (*n* = 6 mice). (I and J) GRAB-DA2h signals (ΔF/F) (60 trials) evoked by photostimulation of the PVN-SNc pathway using 2 mW laser power, 15 minutes after intraperitoneal injection of the DA receptor antagonist haloperidol (Haldol) with 0.4 mg/kg (I) and 1.2 mg/kg (J) (*n* = 6 mice). (K) Statistical graph showing the AUC of GRAB-DA2h signals within 1 second under different photostimulation powers or in the presence of an antagonist (*n* = 60 trials). Data in (K) is expressed as means ± SEM. The broken white lines in the section images represent boundaries of brain regions. Statistical analyses were performed using one-way ANOVA (K) (****P < 0.0001). For *P*-values, see Table S4.

The nigrostriatal pathway, originating in the SNc, densely innervates the DS with dopaminergic neurons. This input is crucial for the normal functioning of the striatum and basal ganglia, which govern voluntary and autonomous movements. Clinically, the depletion of dopaminergic innervation in the striatum is associated with motor deficits, as observed in Parkinson's disease. To monitor dopamine (DA) release in the DS, a genetically encoded GPCR-activation-based dopamine sensor (GRAB-DA sensor) was employed to track changes in DA levels. AAV2/9-C1V1-mCherry and AAV2/9-GRAB-DA2h/DA2h_mut were injected into the PVN and DS, respectively, on the same side in *WT* mice. Optical fibers were implanted above the SNc and DS for fluorescence monitoring (Figure 7A).

Continuous 5-second TNS in TBI mice elevated the GRAB-DA signal in the DS, whereas electrical forelimb stimulation under identical parameters showed no significant change (Figure 7B-C). The viral expression and fiber placement were verified using immunohistochemistry (Figure 7D-E). In TBI mice, SNc neurons with anterograde projections from the PVN were stimulated using a single light pulse (589 nm, 20 ms, 20 Hz, 1-3 mW), resulting in a transient increase in the GRAB-DA signal in the DS. The intensity of the dopamine signal increased proportionally with the power of the light stimulation (Figure 7F-H, K; Figure S5A-C).

As a control, no significant fluorescence changes were observed in DS neurons expressing DA2h_mut (Figure S5D-E). Additionally, the light-induced GRAB-DA signal was abolished by the D2 receptor antagonist haloperidol (Haldol) (Figure 7I-K; Figure S5F-G). These findings confirm that activation of the PVN-SNc-DS pathway triggers DA release in the DS. This supports the hypothesis that TNS restores motor abilities in TBI mice by stimulating DA release through the PVN-SNc-DS pathway.

## Discussion

Assessing the state of arousal in patients with disorders of consciousness is crucial for determining appropriate treatment strategies, planning care, and providing accurate prognostic information 27. This study introduces a novel method to evaluate the state of arousal in TBI mice using a behavioral paradigm based on innate defensive arousal responses to visual looming and auditory stimuli. The findings demonstrate that TNS effectively enhances the defensive arousal levels in TBI mice. These improvements are mediated by distinct neural circuits involving specific neuronal subgroups within the TG.

### TNS reestablishes defensive responses to visual and auditory stimuli in TBI mice

Weight-drop models are widely used to induce mixed focal and diffuse injuries in mice, simulating key aspects of TBI 28. Diffuse axonal injury, a primary feature of TBI, significantly contributes to the variations in arousal levels observed in effected individuals. External impacts on the skull trigger a cascade of pathophysiological changes, including fluctuations in intracranial pressure, subarachnoid and ventricular hemorrhage, brainstem bleeding, prolonged constriction of cerebral microvessels, and insufficient cerebral perfusion. These factors collectively contribute to impairments in the state of arousal 29.

TBI mice demonstrate reduced responsiveness to intense and conspicuous external stimuli, such as visual looming and auditory fear stimuli, characterized by increased response latency and decreased escape speeds. The present study demonstrates that TNS effectively restores defensive arousal responses in TBI mice. Additionally, TNS has been shown to improve motor functions, which may play a role in enhancing escape velocities during defensive reactions. To ascertain whether TNS specifically improves responsiveness to external sensory stimuli in TBI mice, experiments selectively targeted and activated distinct neuronal subgroups within the TG.

### Piezo2+TG neurons and Tac1+TG neurons play distinct roles in rebuilding defensive arousal responses in TBI mice through TNS

The TG houses somatosensory neuron bodies that innervate unique sensory areas of the head and neck, enabling the detection of thermal, mechanical, and chemical signals, thus eliciting various sensory and behavioral responses 30, 31. Advances in single-cell and single-nucleus sequencing technologies have facilitated the classification of distinct neuronal subgroups based on specific gene expression patterns 20, 21. By combining behavioral paradigms with the analysis of activated brain regions, this study identified Piezo2+TG and Tac1+TG neurons as key contributors to the therapeutic effects of TNS.

Activation of Piezo2+TG neurons restored both defensive reactions and motor functions in TBI mice. In contrast, activation of Tac1+TG neurons enhanced defensive responses but did not improve motor abilities. This distinction suggests that Tac1+TG neurons primarily restore sensory sensitivity, whereas Piezo2+TG neurons address both sensory and motor deficits in TBI mice treated with TNS. These findings provide critical insights into the distinct roles of TG neuronal subgroups in mediating the effects of TNS, laying the groundwork for future precision interventions in TBI treatment to target specific neural circuits.

### TNS regenerates sensitivity of the SC receptive field via the Tac1+TG-LC-SC pathway, improving defensive arousal responses to visual looming stimuli in TBI mice

The neural pathways involved in processing visual looming stimuli are well-documented 11. Located just downstream of the retina, the SC is integral to visual information processing and plays a critical role in initiating innate defensive responses to visual threats 32. Through comprehensive viral tracing and experiments involving pathway-specific activation and deactivation, this study highlights the importance of the Tac1+TG-LC-SC pathway in the visual defensive responses of TBI mice. To further elucidate the mechanisms underlying these responses, a behavioral paradigm was developed to isolate visual sensitivity independently of motor skills, enabling precise tracking of SC calcium signals in response to varying speeds of looming stimuli.

Studies have established that low arousal states are associated with hyperpolarization of cortical membrane potentials, resulting in decreased responsiveness to external stimuli and reduced accuracy in task execution 33. In TBI mice, have diminished reactions to varying speeds of looming stimuli were observed. Activation of TNS and the Tac1+TG-LC-SC pathway reversed these deficits. This improvement is likely mediated by LC projections, which modulate cortical and SC activity predominantly through the diffusion of norepinephrine. Supporting this, TNS was shown to induce the release of noradrenaline in the SC via the Tac1+TG-LC-SC pathway.

### TNS reinstates motor abilities in TBI mice by upregulating dopamine levels in the DS through the Piezo2+TG-PVN-SNc-DS pathway, improving defensive arousal responses

Dopaminergic neurons from the SNc interact with the striatum, forming the nigrostriatal pathway, which governs movement through direct and indirect pathways. Research has shown that DA signaling in the DS regulates acceleration and start-stop signals during movement 34. Utilizing viral tracing in combination with pathway-specific activation and deactivation, this study reveals that TNS repairs both motor functions and defensive responses in TBI mice via the Piezo2+TG-PVN-SNc-DS pathway. DS neurons have been identified as synaptic targets within the PVN-SNc pathway, and measurements of DA content changes in the DS confirm that activation of both TNS and the PVN-SNc-DS pathway leads to dopamine release in this region.

Previous research revealed that TNS upregulates DA levels in the hippocampus, contributing to improvements in cognitive function. These findings suggest that the central dopaminergic system plays a crucial role in mediating the effects of TNS. By restoring DA levels in key neural circuits, TNS facilitates recovery of innate defensive arousal responses, motor abilities, and potentially cognitive functions in TBI mice. This highlights the therapeutic potential of targeting dopaminergic pathways for comprehensive recovery in TBI treatment.

DA is recognized as a common regulatory molecule for both the central nervous system and the immune system 35. Numerous immune cells have been shown to express dopamine receptors (DRs) 36, enabling DA to exert immunoregulatory effects in both acute and chronic inflammatory diseases. TBI-induced neuroinflammation manifests as glial cell activation and immune cell infiltration during the acute phase, and as secondary injury, oxidative stress, and blood-brain barrier dysfunction during the chronic phase 37. Neuroinflammation represents a dynamic and complex response. Whether TNS can modulate the inflammatory response in TBI mice and whether the dopaminergic system plays a role in this modulation remain critical questions for further investigation.

This study explores the involvement of two neuronal subgroups within the TG in neural pathways mediating defensive responses to visual stimuli. However, it does not address the neural circuits associated with auditory stimulus sensitivity, highlighting the need for further experiments targeting auditory receptive field sensitivity. Previous studies using monosynaptic retrograde tracing have shown that TG neurons connect to the PVN and LC via indirect pathways 19. This suggests the possibility of neuronal relays occurring in the trigeminal spinal tract nucleus 38 or through alternative routes leading to the downstream PVN and LC 39.

The findings of this study underscore the impact of TNS on enhancing defensive arousal responses in TBI mice, mediated by distinct TG neuronal subgroups through different neural circuits. These insights offer a foundation for the clinical application of TNS as a therapeutic intervention for TBI patients.

## Methods

This study included both male and female animals to assess sex-specific variations in the experimental outcomes. Similar findings were observed across both sexes, indicating no significant sex-based differences in the results.

### Animals

The *Piezo2-*ires-Cre, *Tac1*-ires-Cre, *Trpv1*-Cre, and *Mrgprb4-2A*-Cre mouse lines were obtained from the Jackson Laboratory (JAX Mice and Services). Details regarding the mouse genotypes are provided in Table S1. Mice were housed under controlled environmental conditions, with room temperature maintained at 23 ± 1°C, stable humidity at 50 ± 5%, and a 12 h-hour light/dark cycle. Food and water were provided ad libitum. Mice were housed in groups of 3-5 per cage until they were separated 3 days prior to virus injection. All experimental procedures complied with the animal care and ethical guidelines established by the National Institutes of Health (NIH) and were conducted in accordance with protocols approved by the Animal Care and Use Committee of Sun Yat-sen University.

### TBI model

The TBI model set was established using a modified weight-drop device method 29. Mice were anesthetized with 2% isoflurane and placed on a platform with their ventral side down. A metal rod was dropped onto the exposed skull using a lock mechanical system that ensured accurate determination of the drop height. The left frontotemporal region (2.0 mm lateral to the midline, 1.0 mm anterior to the lambda) was selected as the impact site. A metal rod weighing 30 g was released from a height of 30 cm to induce injury. After the procedure, the scalp wound was sutured, and the mice were returned to their cages for recovery. Mice in the sham group underwent identical surgical procedures, excluding the weight-drop step.

### TNS

After establishing the TBI mouse model, TNS treatment was administered using a low-frequency neuromuscular electrical stimulator. Mice in the TNS group received bilateral electrical stimulation for 60 minutes per session, once daily for two consecutive days. Mice in the TBI group did not receive electrical stimulation. The murine infraorbital nerve was selected as the stimulation target, a feasible site for investigating the neural mechanisms underlying TNS-based neuromodulation therapy. The stimulation location and parameters were slightly adjusted based on previous studies 40.

Mild gas anesthesia was induced using low concentrations of isoflurane (1-1.5%), and facial hair was removed from the mice. Two concentric 27 G surface-mount device (SMD) electrodes (Kedou Brain-Computer Technology, Suzhou, China) were placed parallel to the imaginary line connecting the nose and eyes. Bilateral infraorbital branches of the maxillary nerve (V2) were stimulated transcutaneously. The stimulation parameters were set at a frequency of 40 Hz, a duration of 30 seconds per minute, a current intensity of 0.2 mA, and a pulse width of 200 microseconds. Throughout the stimulation procedure, no significant signs of discomfort were observed in the mice. Mice in the sham group underwent the same procedures, including facial hair removal, electrode attachment, and gas anesthesia, but did not receive actual electrical stimulation.

### Virus vector preparation

Three AAV serotypes-AAV1, AAV2/9, and AAV2-retro—were used in this study. Details of these viral vectors are provided in Table S1. Viral particles were procured from Shanghai Taitool Bioscience Inc. and Wuhan BrainVTA. The initial titers of the viral vectors ranged from 0.8 to 1.5 × 10^13^ particles/ml. For AAV injections, the viral particles were diluted with phosphate-buffered saline (PBS) to achieve a final titer of 2 × 10^12^ viral particles/ml.

### Stereotaxic viral injection

Mice were anesthetized with tribromoethanol (125-250 mg/kg) administered via intraperitoneal injection and were placed on a stereotaxic frame (RWD Life Science, China) for precise surgical procedures. Standard surgeries were performed to expose the brain surface above the TG, SC, LC, PVN, SNc, and DS. Stereotaxic coordinates were used to ensure accurate targeting for each region. For TG injections, the coordinates were bregma -2.10 mm, lateral ±1.90 mm, and dura -5.45 mm. SC injections used coordinates bregma -3.50 mm, lateral ± 0.60 mm, dura -1.15 mm, with an 8° angle from the lateral to medial. LC injection were guided by coordinates bregma -5.25 mm, lateral ± 0.88 mm, and dura -2.95 mm. PVN injections were targeted using bregma -0.41 mm, lateral ± 0.27 mm, and dura -4.63 mm. For SNc injections, the coordinates were bregma -2.90 mm, lateral ± 1.00 mm, and dura -4.20 mm, while DS injections followed coordinates bregma 0.63 mm, lateral ± 2.10 mm, and dura -1.95 mm. AAVs were delivered using a glass pipette connected to a Nanoliter Injector 201 (World Precision Instruments). The injection flow rate was meticulously controlled at 0.15 μl/min to minimize the risk of tissue damage. Following each injection, the pipette was kept in place for a minimum of 20 minutes to allow adequate diffusion of the viral solution and to prevent backflow. Experimental designs related to viral injection are summarized in Table S2.

### Optical fiber implantation and light stimulation

Thirty minutes following AAV injection, a ceramic ferrule equipped with an optical fiber (FC/PC-LC connector: 1.25 mm/2.5 mm; optical fiber core: 200 µm/NA 0.37) was implanted into the target region. The optical fiber tip was positioned at the top of the LC using stereotaxic coordinates (bregma -5.25 mm, lateral ±0.88 mm, dura -2.75 mm) and at the top of the SNc (bregma -2.90 mm, lateral ±1.00 mm, dura -4.00 mm). Once positioned, the ferrule was secured to the skull using dental cement to ensure stability during subsequent experiments. Optogenetic experiments were performed at least three weeks after optical fiber implantation to allow for sufficient expression of the viral constructs. For photogenetic stimulation, the laser output was calibrated and adjusted to intensities of 0.5, 1, 2, and 5 mW before each experiment. The pulse initiation, duration, and frequency of the light stimulation were precisely controlled using a programmable pulse generator connected to the laser system. A 589 nm diode-pumped solid-state laser system (Thinker Tech Nanjing Biotech Co., Ltd) was used to generate the yellow-green laser required for light stimulation. All experiments related to optical fiber implantation are summarized in Table S2.

### Fiber photometry system

Calcium and neurotransmitter signals were recorded using a commercial fiber photometry system (Thinker Tech Nanjing Biotech CO., Ltd). AAV2/9-CaMKIIα-GCaMP6s or AAV2/9-CaMKIIα-EGFP was stereotaxically injected into the SC of *WT* mice. For NE recordings, AAV2/9-hSyn-NE2h or AAV2/9-hSyn-NE_mut was stereotaxically injected into the SC of *WT* mice. For DA recordings, AAV2/9-GRAB-DA2h or AAV2/9-GRAB-DA2h_mut was stereotaxically injected into the DS of *WT* mice.

Following virus injection, an optical fiber (diameter, 230 μm, NA = 0.37; Fiblaser Technology Co., Ltd) was implanted above the SC or DS. Fiber photometry (ThinkerTech) was used to record GCaMP, NE, and GRAB-DA signals. A 488 nm laser beam, reflected by a dichroic mirror and focused by a 10 × lens (NA = 0.3), was coupled to an optical commutator to induce fluorescence signals. A 3-meter optical fiber (200 mm O.D., NA = 0.37) transmitted the light between the commutator and the implanted fiber. To minimize photobleaching, the power intensity at the fiber tip was adjusted to 0.02 mW. Fluorescence signals from the GCaMp6s 41, NE, and GRAB-DA indicators were band-pass filtered (MF525-39, Thorlabs) and collected by a photomultiplier tube (R3896, Hamamatsu). An amplifier (C7319, Hamamatsu) converted the photomultiplier current output into voltage signals, which were further filtered through a low-pass filter (40 Hz cut-off; Brownlee 440). Analog voltage signals were digitalized at 100 Hz and recorded using a Power 1401 digitizer and Spike2 software (CED, Cambridge, UK). Two weeks after AAV injection, fiber photometry was used to record fluorescence signals from GCaMP, NE, and GRAB-DA. Freely moving mice, with optical fibers connected to the photometry system, were allowed to freely explore the arena for 10 minutes. Changes in GCaMP signals in the SC were recorded in response to visual stimuli administered at different rates. Alterations in NE signals in the SC were observed following light stimulation to the LC at varying intensities. Similarly, variations in GRAB-DA signals in the DS were monitored following light stimulation of the SNc at different power levels. Normalized fluorescence changes (ΔF/F) was calculated to measure GCaMP signals and aligned with the onset of visual stimuli. NE and GRAB-DA signals were synchronized to the input time points of light stimulation. After the experiments, the locations of the optical fiber tip were histologically examined for accuracy in each mouse.

### Histological procedure

Mice were anesthetized with urethane and subsequently perfused transcardially with saline, followed by 4% paraformaldehyde (PFA). Brains were carefully removed, post-fixed in 4% PFA overnight, and incubated in PBS containing 30% sucrose until they sank to the bottom of the solution. Coronal sections (40 µm thick) containing the TG, SC, LC, PVN, SNc, or DS were prepared using a cryostat (Leica CM1900). The sections were then incubated in blocking solution (PBS containing 10% goat serum and 0.7% Triton X-100) for 2 hours at room temperature. Following blocking, the sections were treated overnight at 4°C with primary antibodies diluted in the blocking solution. Primary antibodies used for immunohistochemical analysis are displayed in Table S1. The primary antibodies were subsequently removed by washing the sections three times with washing buffer (PBS containing 0.7% Triton X-100). The sections were then incubated with secondary antibodies conjugated with Cy2, Cy3, or Cy5 fluorophores for 2 hours at room temperature. Afterward, the sections were washed three additional times with washing buffer, counterstained with DAPI, and rinsed with PBS. The prepared sections were mounted on Super Frost slides, covered with glass coverslips using mounting medium, and imaged using either a Leica Aperio versa 8 microscope (10× objective lens) or a Zeiss 900 laser scanning confocal microscope (20× and 60× oil-immersion objective lenses). Imaging was performed with sequential laser excitation at 488, 54,6 or 633 nm to prevent signal leakage. The cell-counting strategies are summarized in Table S3. The acquired images, both standard or confocal, were analyzed using the Image J software.

### TG neurons culture

Mice were deeply anesthetized, and the TG were rapidly extracted and placed into D-Hanks solution (Gibco) to maintain tissue viability. Ganglia samples were digested at 37°C in a solution containing collagenase type 2 (2 mg/mL) and Dispase II (7.5 mg/mL) for 30 minutes. The digestion process was halted by suspending the ganglia in 10 mL of DMEM (Gibco) supplemented with 10% bovine calf serum (Gibco). After digestion, the ganglia were mechanically dissociated into a single-cell suspension and plated onto 13 mm glass coverslips pre-coated with poly-D-lysine (100 g/mL, BD Biosciences) to enhance cell attachment. The plated cells were incubated at 37°C in an atmosphere of 5% CO_2_ and 95% O_2_ to promote cell survival and maintenance. Cre-dependent hM3Dq-mCherry labeled TG neurons were used for patch clamp recording within 24 hours of plating to ensure the functional integrity of the cultured neurons.

### TG neuron action potentials recording

For recording action potentials in TG neurons, coverslips containing cultured TG neurons were placed in a 0.5 mL microchamber. The bath solution used during recordings contained (in mM): 145 NaCl, 5 KCl, 2 MgCl2, 2 CaCl2, 10 HEPES, and 10 glucose, with an osmolarity of 320 mOsm and pH adjusted to 7.35 using NaOH. The internal recording solution contained (in mM): 135 K-methanesulfonate, 10 HEPES, 1 EGTA, 1 Na-GTP, and 4 Mg-ATP, with the pH adjusted to 7.35 using KOH, and osmolarity adjusted to 320 mOsm with sucrose. The excitability of TG neurons was assessed in current-clamp mode, recording before and after the administration of CNO (10 Μm in bath solution). To characterize neuronal discharge behavior, current injections were applied in 20 pA increments, starting at 0 pA and increasing up to 100 pA. Each step lasted 300 ms. These protocols enabled the precise characterization of TG neuron action potential patterns and their responsiveness to pharmacological stimulation with CNO.

### Preparation of behavioral tests

Following AAV injection and optical fiber implantation, mice were housed individually for three weeks to allow for recovery and sufficient viral expression. Mice were handled daily by the experimenters for at least three days before the behavioral tests to minimize stress and ensure acclimation to human interaction. On the day of behavioral testing, mouse cages were relocated to the testing room, where the mice were habituated to the room's conditions for three hours prior to start of the experiments. The testing apparatus was thoroughly cleaned with 20% ethanol between trials to eliminate odor cues left by other mice, ensuring unbiased results. All behavioral tests were performed during the same circadian period, between 1 PM and 7 PM, to maintain consistency in experimental conditions. Behavioral scoring was conducted by experimenters who were blinded to the treatment groups to eliminate potential observer bias and ensure objective data collection.

### Neurological severity score (NSS)

To ensure a consistent baseline severity of injury after TBI modeling, the NSS was utilized to assess functional neurological impairments post-TBI 42. The score consists of ten separate parameters, including motor function, alertness, and physiological behavior tasks. A point was awarded if there was a lack of test reflexes or an inability to perform a task, with no points given for successful completion. Thus, a maximum score of 10 on the NSS indicates severe neurological impairment, with failure in all tasks. Each NSS task is described as follows.

Task 1: Exit circle. A mouse was placed in the center of the circle and the time needed for the mouse to exit the apparatus was monitored. A healthy mouse, as it has an intrinsic seeking behavior, usually leaves the circle within 3 minutes. If the mouse was still inside the circle after 3 minutes, 1 point was given; otherwise, 0 points were counted.

Task 2: Seeking behavior. Again, a mouse was placed in the center of the circle, but the exit was closed. The ability of the mouse to explore the environment and exhibit sniffing behavior within 3 minutes was monitored. When the mouse did not perform seeking behavior, 1 point was counted; if seeking behavior was observed, 0 points were counted.

Task 3: Monoparesis/hemiparesis. Anatomic forceps were used to assess grip strength. Mice were held by the tail, and their paws were touched with the forceps. A score of 0 points was given if the mouse gripped the forceps, and 1 point was added if the mouse failed to grip or displayed very low grip intensity.

Task 4: Straight walk. A mouse was placed on a flat surface to evaluate its ability to walk straight and its alertness. If the mouse demonstrated an impaired gait pattern or failed to actively explore its environment, 1 point was assigned. A normal walking pattern resulted in a score of 0 points.

Task 5: Startle reflex. A loud clap was used to elicit a startle reflex. If the mouse displayed a reaction, 0 points were assigned; if no reaction was observed, 1 point was given.

Task 6: Beam balance. The mouse's ability to balance on the beam (0.5 × 0.5 mm2) for 10 seconds was evaluated. A successful balance resulted in a score of 0 points, while failure to balance was scored as 1 point.

Tasks 7, 8, 9: Beam walk. A mouse was placed at one end of beams with widths of 3 cm, 2 cm, and 1 cm, each 30 cm in length, to evaluate its intrinsic seeking reflex and ability to cross. If the mouse crossed the beams and reached the opposite end within 3 minutes, 0 points were given. If it failed to cross or reach the other side within 3 minutes, 1 point was assigned for each beam. If the mouse failed to cross the 3-cm-wide beam, the narrower 2-cm and 1-cm beam tasks were not conducted, and 3 or 2 points were given, respectively.

Task 10: Round stick balance. The mouse was tested for its ability to balance on a round stick of 5 mm in diameter for 10 seconds. Successful balancing resulted in a score of 0 points, while failure was assigned 1 point.

The total NSS was calculated by summing the points from all ten tasks, providing a comprehensive assessment of the degree of neurological impairment. The NSS assessed 1 hour post-TBI reflects the initial severity of the closed head injury, with strong correlations observed between NSS values, magnetic resonance imaging data, and histological findings. This correlation indicates that NSS accurately represents the extent of brain tissue damage caused by TBI. For this study, mice scoring between 5 and 8 were selected, as these scores indicate a comparable severity of injury. Selected mice were randomly assigned to either the TBI group or the TBI+TNS group to ensure balanced experimental conditions. Mice scoring above 8 were excluded from the study in accordance with protocol guidelines to prevent extreme suffering and reduce high mortality rates.

### Visual looming stimuli

Defensive behaviors triggered by visual looming stimuli were assessed in a behavioral box measuring 35 cm × 35 cm, lined with standard mouse bedding. A shelter was placed in one corner of the box to provide an optional hiding space. A conventional computer monitor was positioned overhead to deliver visual looming stimuli, which appeared as a continuously expanding dark disk on a light background. The luminance of the dark disk and background was set at 0.1 and 3.6 cd/m², respectively. Mice were introduced to the box and allowed to explore freely for 10 minutes. Following the acclimation period, three cycles of visual looming stimuli were presented. Mouse behavior was recorded using two orthogonally positioned cameras operating at 25 frames per second (fps), equipped with infrared LED illumination to enable nighttime-like observations. The latency of the escape response was defined as the time interval between the onset of the visual stimulus and the initiation of head-turning movements or acceleration. For mice that did not exhibit an escape response, a latency value of 5000 ms was assigned for statistical analysis. The escape velocity was measured using the VisuTrack-AI animal behavior analysis software (Shanghai Xinsoft), which allowed real-time tracking of changes in movement speed and provided measurements of peak escape velocity during the stimulus. Instantaneous movement speed was calculated in 50 ms intervals.

Prior to TBI modeling, a sensory stimulus screening was conducted to select mice with comparable escape response times and speeds, ensuring consistency across experimental groups. Previous studies have demonstrated that repeated exposure to visual looming stimuli on the same day can lead to learned suppression of escape behaviors 43. To prevent habituation, only one visual stimulus was applied per experimental session. Preliminary experiments showed that mice with significant escape responses could replicate these behaviors two and three days after the initial stimulus without evidence of habituation or memory effects.

### Auditory fear stimuli

Auditory fear responses were assessed using an ultrasonic speaker positioned 15 cm above the behavioral box, with a digital camera mounted overhead to continuously record mouse activity. The auditory stimulus consisted of a sound that gradually increased in frequency from 17 kHz to 20 kHz over 100 milliseconds, cycling for a total duration of 2 seconds to constitute one auditory fear stimulus. At the start of each test, mice were transferred from their home cages to the behavioral box and allowed to explore freely for 10 minutes. When a mouse entered the central area of the box, the experimenter manually triggered the sound stimulus via a computer. The sound, amplified to 70 dB and ranging from 17 to 20 kHz, was delivered as stimulus. A 10-minute interval was maintained between each stimulus to minimize habituation, and the defensive responses elicited by the first stimulus were analyzed for statistical purposes. Two parameters were extracted from the recorded video footage to quantify the defensive reactions: latency and speed of the escape response. Latency was defined as the time interval from the onset of the sound stimulus to the initiation of head-turning movements or acceleration. For mice that did not exhibit an escape response, a latency value of 5000 ms was assigned for statistical analysis. The escape velocity was calculated using VisuTrack-AI animal behavior analysis software (Shanghai Xinsoft), which provided real-time measurements of changes in mouse movement speed and determined the peak escape velocity during the stimulus. Instantaneous movement speeds were calculated at 50 millisecond intervals.

### Visual stimuli at varying rates

Retinal signals form spatially topographic maps within the SC. Inputs from the right visual field project to the left SC, and inputs from the left visual field project to the right SC. The nasal-to-temporal axis is represented along the anterior-to-posterior direction, while the upper-to-lower axis is represented medial to laterally 44. This organizational framework is conserved across vertebrates, including humans. Since visual looming threats typically originate from above the mice, recordings were conducted from optic fibers embedded in the medial SC to capture responses from this region.

Visual stimuli were presented using a cathode-ray tube (CRT) monitor with a 60 Hz refresh rate and an average luminance of ~35 cd/m^2^, positioned opposite the recording hemisphere. During the experiments, only the contralateral eye of the mouse was stimulated, while the ipsilateral eye was covered with a light blocker to isolate responses. To determine the mouse's most sensitive visual field position, the monitor was divided into 15 regions. Looming stimuli at a speed of 80 °/s were presented in each region, and the location eliciting the most pronounced GCaMP signal response was selected. This region was typically located along the first row of the monitor. Once the optimal visual field position was identified, the distance and angle between the mouse and the screen were fixed. Each stimulus was repeated five times in a pseudorandom order, with a one-second interval between conditions. Blank stimuli (gray background) were included to measure spontaneous activity as a control. Black looming circles (~35 cd/m^2^) then appeared at varying speeds at the center of the unit's receptive field, displayed against the gray background. The final diameter of the looming circles was fixed at 40°.

Two weeks after virus injection and fiber implantation, a behavioral paradigm was implemented to assess visual stimulus sensitivity curves at varying rates, establishing baseline visual stimulus response curves for each mouse. Following TBI modeling, the mice were randomly assigned to either a TBI group or a TBI+TNS treatment group. The TBI+TNS group received continuous TNS treatment for two consecutive days. GCaMP signals in response to different visual stimulus rates were recorded on the first and second days post-TBI. The ΔF/F (%) values at each looming rate were calculated as the average of five peak values from the GCaMP signal waveforms, providing a quantitative measure of visual sensitivity. Data inclusion was contingent on histological verification of correct virus injection locations and proper fiber placements.

### Rotarod fatigue test

The rotarod fatigue test utilized a balance beam apparatus consisting of a 40 cm-long beam with extensions at both ends, elevated 30 cm above the ground. One end of the beam was associated with aversive stimuli, including intense light and noise, while the other end led to a dark, enclosed box. This setup leveraged the natural aversion and exploratory behaviors of rodents to motivate them to traverse the beam. The time taken by the mouse to cross the beam was recorded and served as an indicator of motor coordination and the extent of forelimb and hindlimb injuries.

### Balance beam test

To prepare for the balance beam test, the mice underwent daily 30-minute training sessions on the rotarod during the week preceding the experiment. During training, if a mouse fell off the rod, it was immediately placed back onto it to acclimate to the apparatus. To ensure reliable motor function assessments, animals were allowed at least 24 hours of recovery post-surgery to eliminate the influence of anesthetic agents. Before the test, each mouse was trained on the rotarod at a constant slow speed (e.g., 5 rpm) for 5 minutes. Following this acclimation, a 1-hour interval was provided before initiating the trials. During the test, the rotarod was programmed to accelerate progressively (e.g., starting at 4 rpm and increasing at 20 rpm/min up to a maximum speed of 40 rpm). Mice were positioned on the rod facing away from the direction of rotation, requiring them to walk forward to maintain their position. The trial began with the activation of the motor and timer, and it was considered complete when the mouse fell from the rod. The latency to fall and the rotational speed at the time of falling were recorded for each trial. This process was repeated three times for each mouse, with a 15-minute interval between trials. The average latency to fall or average rotational speed across the three trials was calculated and used as an indicator of motor function for subsequent analyses. To ensure hygiene and prevent cross-contamination, the apparatus was cleaned and disinfected between tests for each mouse.

### Open field test

The open field test assessed a mouse's behavioral performance in an unfamiliar environment, evaluating spontaneous activity, exploratory behaviors, and anxiety levels. This method provided insights into autonomous behaviors, exploratory patterns, and emotional responses in novel settings. Additional parameters, such as total distance traveled, movement speed, and resting time, were analyzed to quantify behavioral performance. The open field test box measured 50 cm × 50 cm × 40 cm and was constructed from white, single-sided matte acrylic to minimize reflections on the inner walls. The VisuTrack-AI animal behavior analysis software (Shanghai Xinsoft) was used to automatically track the mouse's movement trajectory, identify its center of gravity, and record total travel distance and average speed. Before the experiment, animals were allowed to acclimate to the testing environment for 30 to 60 minutes. Once acclimated, each mouse was placed in the center of the test box, consistently introduced from the same position and direction for each trial. Monitoring began immediately and continued for 10 minutes. Evaluation metrics include total distance traveled, average movement speed, and total movement time.

### Quantification and statistical analysis

Data were expressed as mean ± SEM and analyzed using Graphpad Prism (version 9, GraphPad Software, San Diego, CA, USA). To compare two groups, unpaired two-tailed Student's t-test was used. For comparisons involving multiple groups, one-way or two-way ANOVA was employed depending on the experimental design. A P value of less than 0.05 was considered statistically significant. Detailed information on statistical analyses is provided in the figure legends and Table S4.

## Supplementary Material

Supplementary figures and tables.

## Abbreviations

TNS: trigeminal nerve stimulation
TBI: traumatic brain injury
TG: trigeminal ganglion
LC: locus coeruleus
SC: superior colliculus
PVN: paraventricular hypothalamic nucleus
SNc: substantia nigra pars compacta
DS: dorsal striatum
sSC: superficial SC
LP: lateral posterior nucleus
PV: parvalbumin
PBG: parabigeminal nucleus
m/dSC: intermediate/deep SC
IC: inferior colliculus
ACx: auditory cortex
NSS: neurological severity score
AAV: adeno-associated virus
CNO: clozapine N-oxide
WT: wild-type
NE: noradrenaline
YO: yohimbine
RV: rabies virus
SS: somatosensory area
ILM: intralaminar nuclei of the dorsal thalamus
PRV: pseudorabies virus
GRAB-DA: GPCR-activation-based dopamine
Haldol: haloperidol
DRs: dopamine receptors
