# Supplementary material for: Neural mechanism of trigeminal nerve stimulation recovering defensive arousal responses in traumatic brain injury
Source: Theranostics. 2025 Jan 13;15(6):2315–37. doi: 10.7150/thno.106323 (PMC11840743; doi:10.7150/thno.106323)
Supplement: Supplementary file 1 — Supplementary figures and tables. [file thnov15p2315s1.pdf]

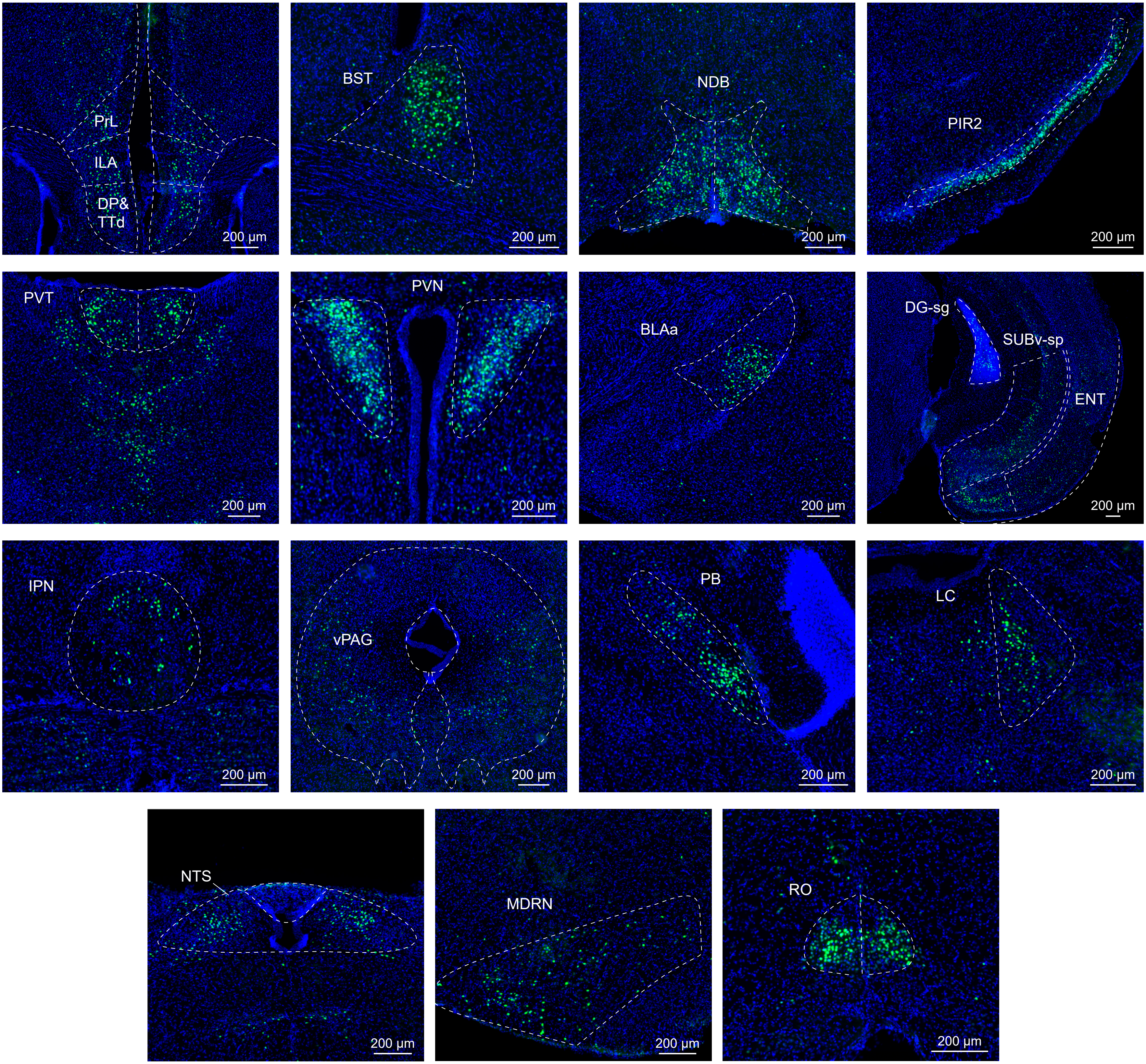


**Figure S1. Coronal brain sections showing increased c-Fos expression in brain regions following TNS stimulation (Related to Figure.1).** The broken white lines in the section images represent boundaries of brain regions. PrL, prelimbic cortex; ILA, infralimbic area; DP, dorsal peduncular cortex; TTd, taenia tecta, dorsal part; BST, bed nucleus of the stria terminalis; NDB, diagonal band nucleus; PIR2, piriform area, pyramidal layer; PVT, paraventricular thalamic nucleus; PVN, paraventricular hypothalamic nucleus; BLAa, basolateral amygdaloid nucleus, anterior part; DG-sg, dentate gyrus, granule cell layer; SUBv-sg, subiculum, ventral part, pyramidal layer; ENT, entorhinal area; IPN, interpeduncular nucleus; vPAG, periaqueductal gray, ventral part; PB, parabrachial nucleus; LC, locus coeruleus; NTS, nucleus of the solitary tract; MDRN, medullary reticular nucleus; RO, raphe obscurus nucleus.

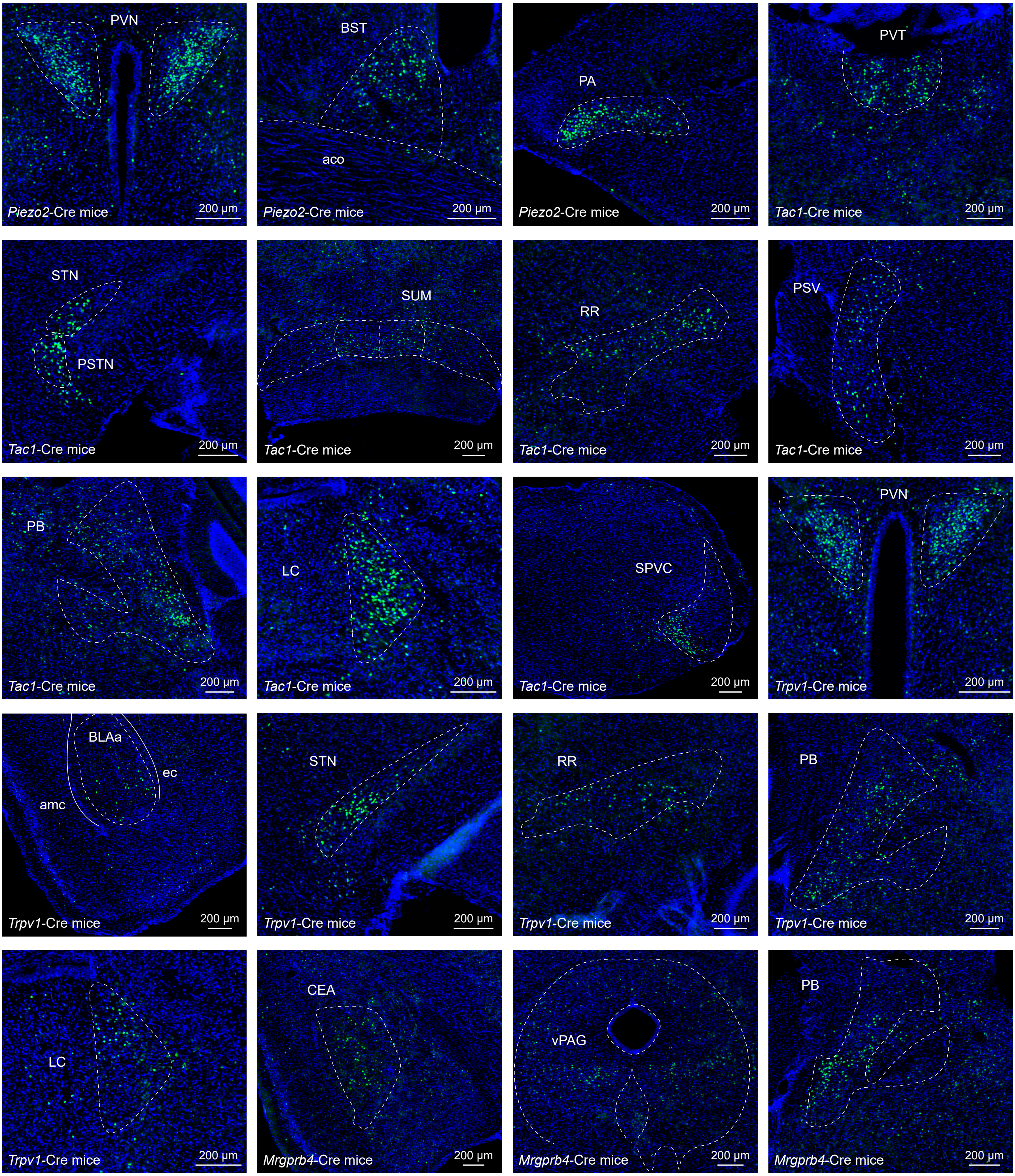


**Figure S2. Coronal brain sections illustrating regions of increased c-Fos expression following chemogenetic activation of bilateral TG in *Piezo2*-Cre, *Tac1*-Cre, *Trpv1*-Cre, and *Mrgprb4*-Cre mice (Related to Figure 2).** The broken white lines in the section images represent boundaries of brain regions. PVN, paraventricular hypothalamic nucleus; BST, bed nucleus of the stria terminalis; aco, anterior commissure, olfactory limb; PA, posterior amygdalar nucleus; PVT, paraventricular thalamic nucleus; STN, subthalamic nucleus; PSTN, parasubthalamic nucleus; SUM, supramammillary nucleus; RR, retrorubral nucleus; PSV, principal sensory nucleus of the trigeminal; PB, parabrachial nucleus; LC, locus coeruleus; SPVC, spinal trigeminal nucleus, caudal part; BLAa, basolateral amygdalar nucleus, anterior part; amc, amygdalar capsule; ec, external capsule; CEA, central amygdaloid nucleus; vPAG, periaqueductal gray, ventral part.

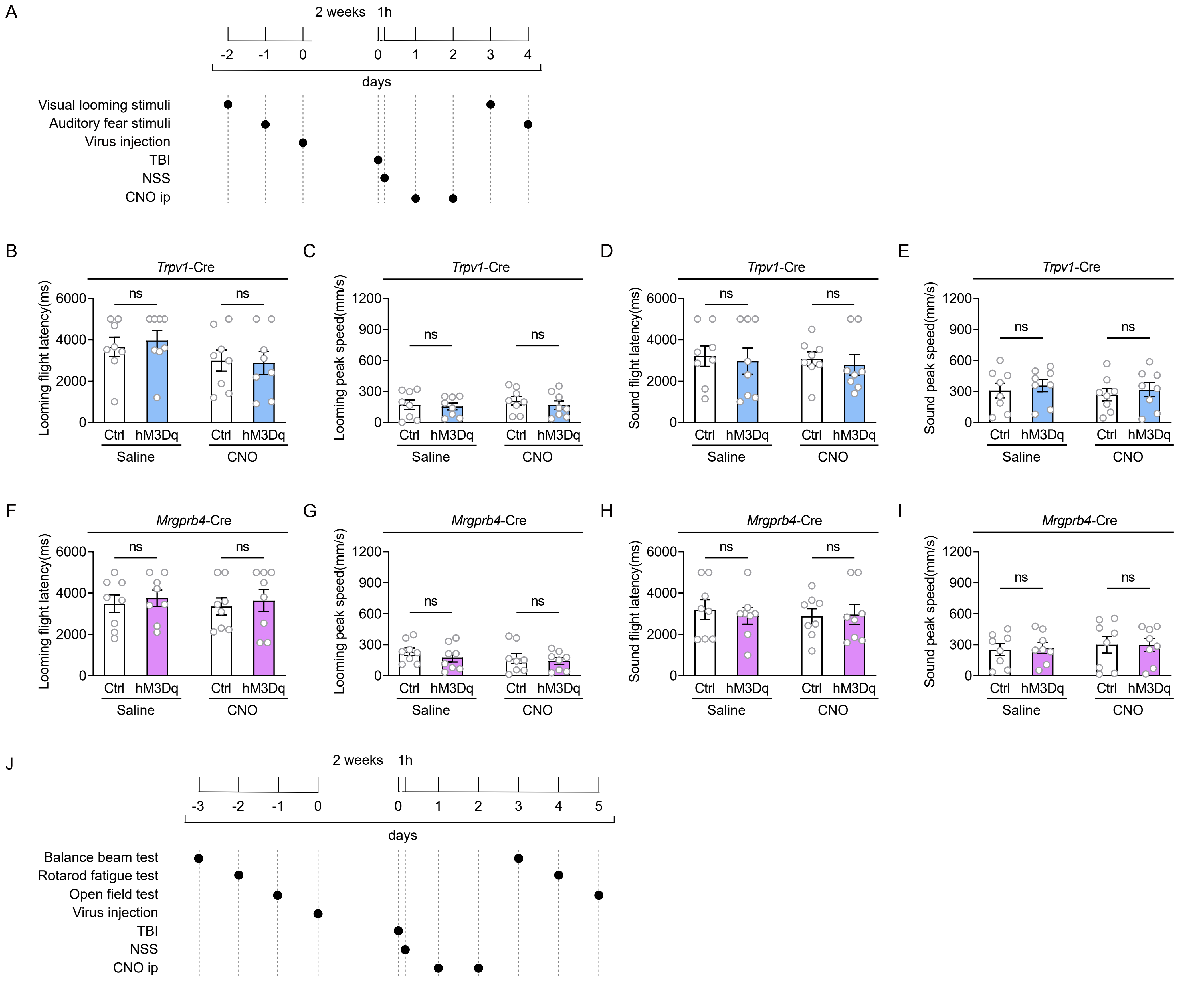


**Figure S3. Chemogenetic activation of subgroups of TG neurons (Related to Figure 2).** (**A**) Schematic diagram showing experimental design for defensive arousal response in chemogenetically evoked *Piezo2*-Cre, *Tac1*-Cre, *Trpv1*-Cre, and *Mrgprb4*-Cre mice after TBI. (**B** and **C**) Latency (**B**) and peak escape velocity (**C**) of the visual looming defensive response in TBI mice with and without chemogenetic activation of Trpv1+ TG neurons ($n$ = 8 mice). (**D** and **E**) Latency (**D**) and peak escape velocity (**E**) of the auditory stimulus defensive response in TBI mice with and without chemogenetic activation of Trpv1+ TG neurons ($n$ = 8 mice). (**F** and **G**) Latency (**F**) and peak escape velocity (**G**) of the visual looming defensive response in TBI mice with and without chemogenetic activation of Mrgprb4+ TG neurons ($n$ = 8 mice). (**H** and **I**) Latency (**H**) and peak escape velocity (**I**) of the auditory stimulus defensive response in TBI mice with and without chemogenetic activation of Mrgprb4+ TG neurons ($n$ = 8 mice). (**J**) Schematic diagram showing experimental design for motor ability in chemogenetically evoked *Piezo2*-Cre and *Tac1*-Cre mice after TBI. Data in (**B-I**) are expressed as means ± SEM. Statistical analyses were performed using Student *t*-tests (**B-I**) (ns, not significant, $P > 0.05$). For *P*-values, see Table S4.

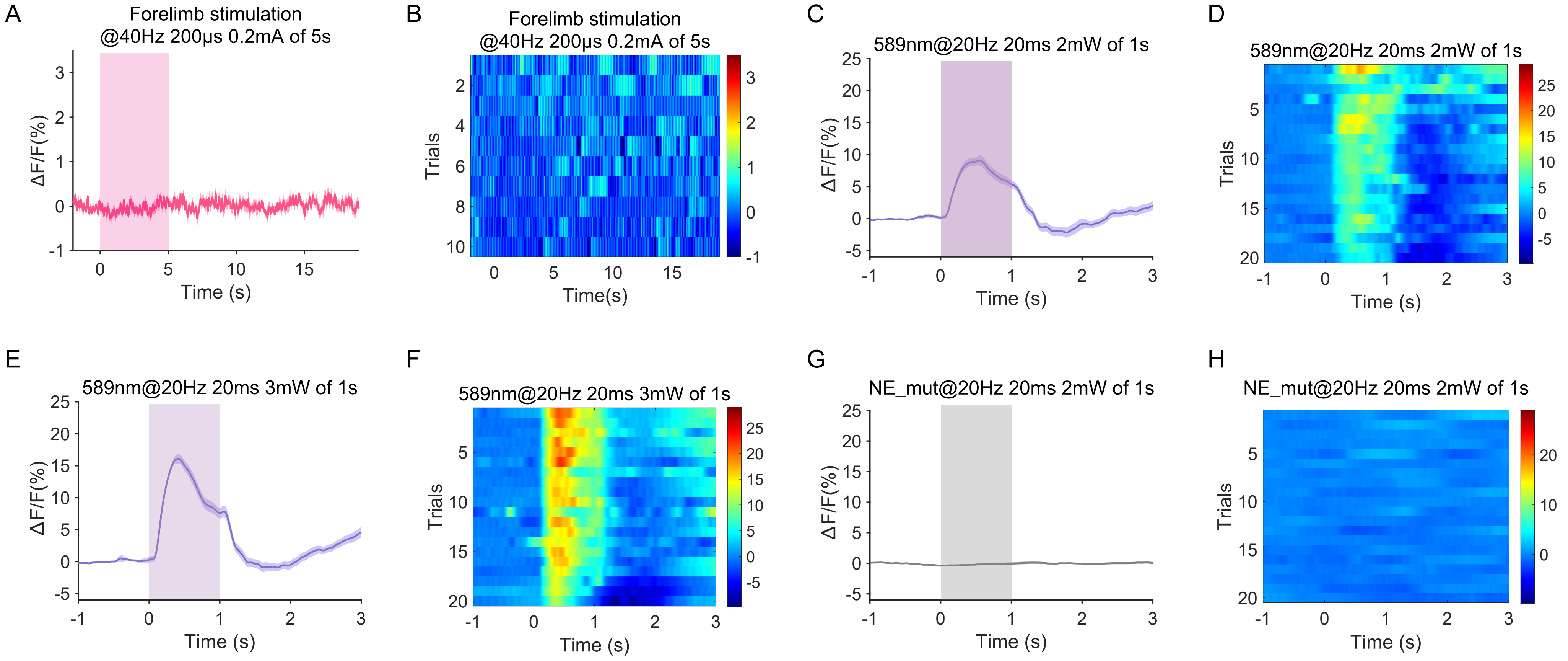


**Figure S4. Variations in NE levels in the SC during electrical stimulation of the forelimbs and various intensities of LC-SC pathway activation (Related to Figure 5).** (**A** and **B**) NE signals (ΔF/F) (**A**) and heat map (**B**) of 10 trials in the SC during electrical stimulation of mouse forelimbs (stimulation parameters identical to TNS: 200 μs, 0.2 mA, 40 Hz, 5 seconds) (*n* = 5 mice). (**C** and **D**) NE signals (ΔF/F) (**C**) and heat map (**D**) of 20 trials evoked by photostimulation of TH+ LC neurons with 2 mW laser power (589 nm, 20 ms, 20 Hz, 1 pulse of 1 second) (*n* = 5 mice). (**E** and **F**) NE signals (ΔF/F) (**E**) and heat map (**F**) of 20 trials evoked by photostimulation of TH+ LC neurons with 3 mW laser power (589 nm, 20 ms, 20 Hz, 1 pulse of 1 second) (*n* = 5 mice). (**G** and **H**) NE signals (ΔF/F) (**G**) and heat map (**H**) of 20 trials evoked by photostimulation of TH+ LC neurons in NE_mut control mice (*n* = 5 mice).

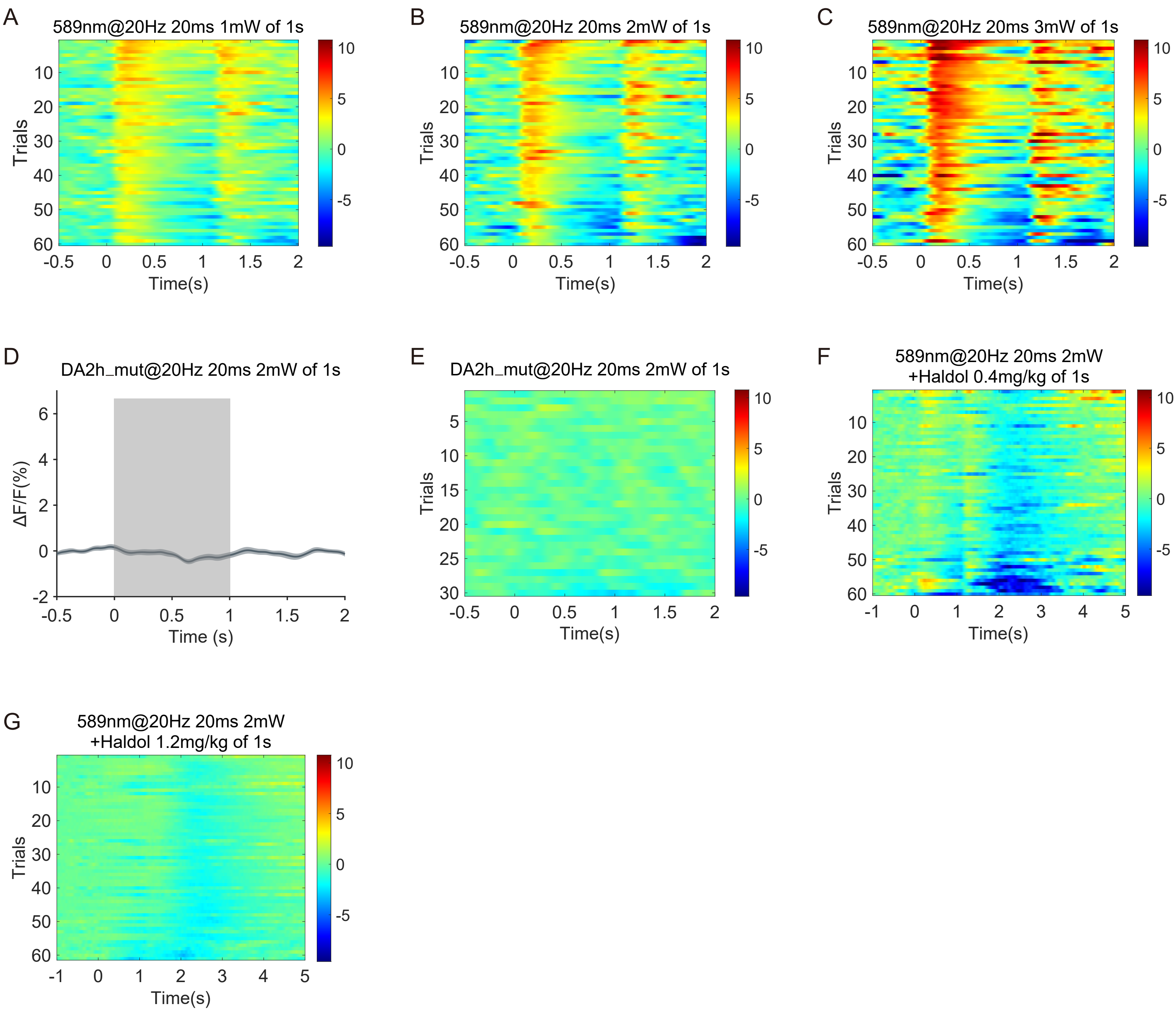


**Figure S5. Variations in GRAB-DA2h signal with varying intensities of PVN-SNc pathway activation, compared to DA2h_mut control and following antagonist application (Related to Figure 7).** (**A-C**) Heat maps (60 trials) of GRAB-DA2h signals in the DS during activation of the PVN-SNc pathway with different photostimulation intensities (1 mW (**A**), 2 mW (**B**), and 3 mW (**C**)) (*n* = 6 mice). (**D** and **E**) GRAB-DA2h signals (ΔF/F) (**D**) and heat maps (**E**) of 30 trials in the DS of DA2h_mut control mice when the PVN-SNc pathway is stimulated with 2mW laser power (*n* = 6 mice). (**F** and **G**) Heat maps (60 trials) of GRAB-DA2h signals evoked by photostimulation of the PVN-SNc pathway using 2 mW laser power, 15 minutes after intraperitoneal injection of the DA receptor antagonist haloperidol (Haldol) with 0.4 mg/kg (**F**) and 1.2 mg/kg (**G**) (*n* = 6 mice).

**Table S1. Information of mouse lines and reagents**

| **Mouse Lines** | | |
|---|---|---|
| *Piezo2*-ires-Cre | JAX Mice | Stock No: 027719 |
| *Tac1*-ires-Cre | JAX Mice | Stock No: 021877 |
| *Trpv1*-Cre | JAX Mice | Stock No: 017769 |
| *Mrgprb4*-2A-Cre | JAX Mice | Stock No: 021077 |
| *TH-Cre* | JAX Mice | Stock No: 008601 |
| *DAT*-ires-*Cre* | JAX Mice | Stock No: 006660 |
| *C57BL/6J* | Sun Yat-sen University Laboratory Animal Center | N/A |
| **Antibodies** | | |
| Rabbit polyclonal anti-EGFP | Abcam | ab290 |
| Chicken polyclonal anti-EGFP | Abcam | ab13970 |
| Chicken polyclonal anti-mCherry | Abcam | ab205402 |
| Rabbit monoclonal Anti-Fos | Cell Signaling Technology | 2250 |
| Rabbit polyclonal anti-NeuN | Abcam | ab177487 |
| Rabbit recombinant Anti-Tyrosine Hydroxylase | Abcam | ab137869 |
| Goat anti-Rabbit IgG (H+L) Alexa 488 | Thermo Fisher Scientific | A11034 |
| Goat anti-Chicken IgY (H+L) Alexa 488 | Thermo Fisher Scientific | A11039 |
| Goat anti-Rabbit IgG (H+L) Alexa 546 | Thermo Fisher Scientific | A11010 |
| Goat anti-Chicken IgY (H+L) Alexa 546 | Thermo Fisher Scientific | A11040 |
| Goat anti-Mouse IgG (H+L) Alexa 546 | Thermo Fisher Scientific | A11030 |
| Goat anti-Mouse IgG (H+L) Alexa 647 | Thermo Fisher Scientific | A21235 |

| Chemicals, peptides, and recombinant proteins | | |
|---|---|---|
| Clozapine N-oxide | ENZO | BML-NS105 |
| DAPI | Solarbio | C0065 |
| Dispase® II | Roche | 04942078001 |
| Collagenase type 2 | Worthington-biochem | LS004176 |
| Yohimbine | Macklin | Y820631 |
| Haloperidol hydrochloride | Aladdin | H275922 |
| **Bacterial and virus strains** | | |
| AAV2/9-hSyn-DIO-hM3Dq-mCherry | TaiToll | N/A |
| AAV2/9-hSyn-DIO-mCherry | TaiToll | N/A |
| AAV2/9-hSyn-DIO-EGFP | TaiToll | N/A |
| AAV2-retro-hSyn-DIO-Flp | BrainVTA | N/A |
| AAV2/9-hSyn-fDIO-hM3Dq-EGFP | BrainVTA | N/A |
| AAV2/9-hSyn-taCasp3-TEVp-EGFP | BrainVTA | N/A |
| AAV2/9-hSyn-EGFP | BrainVTA | N/A |
| AAV2/9-CaMKIIα-GCamp6s | BrainVTA | N/A |
| AAV2/9-hSyn-NE2h | BrainVTA | N/A |
| AAV2/9-hSyn-NE_mut | BrainVTA | N/A |
| AAV2/9-hSyn-DIO-C1V1-mCherry | BrainVTA | N/A |
| AAV2/9-EF1α-DIO-H2B-EGFP-T2A-TVA | BrainVTA | N/A |
| AAV2/9-EF1α-DIO-RVG | BrainVTA | N/A |
| RV-EnvA-∆G-DsRed | BrainVTA | N/A |
| PRV-CAG-EGFP | BrainVTA | N/A |

| | | |
|---|---|---|
| AAV2-retro-hSyn-Cre | BrainVTA | N/A |
| AAV1-hSyn-Flp | BrainVTA | N/A |
| AAV2/9-hSyn Con&Fon-hM3Dq-mCherry | BrainVTA | N/A |
| AAV2/9-hSyn Con&Fon-mCherry | BrainVTA | N/A |
| AAV2/9-hSyn-GRABeen DA2h | TaiToll | N/A |
| AAV2/9-hSyn-GRABeen DA2h_mut | TaiToll | N/A |
| AAV2/9-hSyn-C1V1-mCherry | BrainVTA | N/A |

**Table S2. Summary of all experimental designs**

| Figures | Aims | Mouse lines | AAV injection & optical fiber implantation | Type of data |
|---|---|---|---|---|
| Fig.1A-1I | Clarifying the role of TNS in defensive arousal in mice with TBI | *WT* | No AAV injection.<br>No optical fiber implantation. | Behavior |
| Fig.1J-1N | Assessing the impact of TNS on motor abilities in mice with TBI | *WT* | No AAV injection.<br>No optical fiber implantation. | Behavior |
| Fig. S1 | Identify nuclei associated with TNS | *WT* | No AAV injection.<br>No optical fiber implantation. | Histology |
| Fig.2A-2Q | Activation various TG neurons subtypes | *Piezo2*-Cre<br>*Tac1*-Cre<br>*Trpv1*-Cre<br>*Mrgprb4*-Cre | AAV2/9-hSyn-DIO-hM3Dq-mCherry injected into the TG. | Behavior<br>Slice physiology<br>Histology |
| Fig.S2 | Identify nuclei associated with the activation of distinct TG neuron subpopulations | *Piezo2*-Cre<br>*Tac1*-Cre<br>*Trpv1*-Cre<br>*Mrgprb4*-Cre | AAV2/9-hSyn-DIO-hM3Dq-mCherry or AAV2/9-hSyn-DIO-mCherry injected into the TG. | Histology |
| Fig.S3 | Activation various TG neurons subtypes | *Trpv1*-Cre<br>*Mrgprb4*-Cre | AAV2/9-hSyn-DIO-hM3Dq-mCherry or AAV2/9-hSyn-DIO-mCherry injected into the TG. | Behavior |
| Fig.3A-3B | Anterograde tracing of TH+ LC neurons | *TH*-Cre | AAV2/9-hSyn-DIO-EGFP injected into the LC. | Histology |
| Fig.3C-3G | Activation of SC-projecting TH+ LC neurons | *TH*-Cre | AAV2-retro-DIO-Flp injected into the SC.<br>AAV2/9-fDIO-hM3Dq-EGFP or AAV-fDIO-EGFP injected into the LC. | Behavior<br>Histology<br>Slice physiology |
| Fig.3G-3N | Partial ablation of LC while activating Tac1+ TG | *Tac1*-Cre | AAV2/9-hSyn-taCasp3-TEVp-EGFP or AAV2/9-hSyn-EGFP | Behavior<br>Histology |

| | | | | |
|---|---|---|---|---|
| | | | injected into the LC.<br>AAV2/9-hSyn-DIO-hM3Dq-mCherry injected into the TG. | |
| Fig.4A-4G | Recording GCaMP signals in response to visual stimuli at various rates | *WT* | AAV2/9-CaMKIIα-GCaMP6s injected into the SC.<br>Optical fiber implanted above the SC. | Behavior<br>Histology<br>Fiber photometry |
| Fig.4H-4L | Recording GCaMP signals in response to visual stimuli at various rates after TNS treatment | *WT* | AAV2/9-CaMKIIα-GCaMP6s injected into the SC.<br>Optical fiber implanted above the SC. | Behavior<br>Histology<br>Fiber photometry |
| Fig.4M-4R | Recording GCaMP signals in response to visual stimuli at various rates after LC activation | *TH*-Cre | AAV2/9-CaMKIIα-GCaMP6s injected into the SC.<br>AAV2/9-hSyn-DIO-hM3Dq-mCherry or AAV2/9-hSyn-DIO-mCherry injected into the LC.<br>Optical fiber implanted above the SC. | Behavior<br>Histology<br>Fiber photometry |
| Fig.5A-5C<br>Fig.S4A-S4B | Recording NE signals during TNS | *WT* | AAV2/9-hSyn-NE2h injected into the SC.<br>Optical fiber implanted above the SC. | Histology<br>Fiber photometry |
| Fig.5D-5M<br>Fig.S4C-S4H | Recording NE signals during the activation of TH+ LC | *TH*-Cre | AAV2/9-hSyn-NE2h or AAV2/9-hSyn-NE_mut injected into the SC.<br>AAV2/9-DIO-C1V1-mCherry injected into the LC.<br>Optical fiber implanted above the SC and LC. | Histology<br>Fiber photometry |
| Fig.6A-6E | Retrograde tracing of DS-SNc pathway | *DAT*-Cre | AAV-DIO-EGFP-T2A-TVA & AAV-DIO-RVG injected into the SNc.<br>RV-EnvA-ΔG-DsRed injected into the DS. | Histology |
| Fig.6F- | Retrograde tracing of DS | *WT* | PRV-CAG-EGFP injected into the DS. | Histology |
| Fig.6G- | Activation of SNc | *WT* | AAV2-retro-Cre | Behavior |

| | | | | |
|---|---|---|---|---|
| 6J | neurons receiving anterograde projections from the PVN and retrograde projections from the DS | | injected into the DS.<br>AAV1-Flp injected into the PVN.<br>AAV2/9-hSyn Con&Fon-hM3Dq-EGFP or AAV2/9-hSyn Con&Fon-EGFP injected into the SNc. | Histology<br>Slice physiology |
| Fig.6K-6Q | Partial ablation of PVN while activating Piezo2+ TG | *Piezo2*-Cre | AAV2/9-hSyn-taCasp3-TEVp-EGFP or AAV2/9-hSyn-EGFP injected into the PVN.<br>AAV2/9-hSyn-DIO-hM3Dq-mCherry injected into the TG. | Behavior<br>Histology |
| Fig.7A-7C | Recording GRAB-DA signals during TNS | *WT* | AAV2/9-hSyn-GRABeen DA2h injected into the DS.<br>Optical fiber implanted above the DS. | Histology<br>Fiber photometry |
| Fig.7D-7K<br>Fig.S5A-S5G | Recording GRAB-DA signals during the activation of PVN-SNc pathway | *WT* | AAV2/9-hSyn-GRABeen DA2h or AAV2/9-hSyn-EGFP injected into the DS.<br>AAV2/9-C1V1-mCherry injected into the PVN.<br>Optical fiber implanted above the SNc and DS. | Histology<br>Fiber photometry |

**Table S3. Summary of Cell Counting Strategy**

| Brain region | Section type | Section Range | Total collection | Sampling |
|---|---|---|---|---|
| LC | Coronal section (40 μm) | Bregma (-5.28 to -5.68) | Approximately 5 sections | Sampling 1 section every 2 sections to obtain 5 sections evenly spaced by 80 μm. |
| PVN | Coronal section (40 μm) | Bregma (-0.58 to -1.20) | Approximately 5 sections | To sample 1 section every 3 sections to get 5 sections evenly spaced by 120 μm |
| The whole brain | Coronal section (40 μm) | The whole brain | Approximately 300 sections | To sample 1 section every 5 sections to get 50 sections evenly spaced by the whole brain |

**Table S4. Summary of statistical analyses**

| Figure | Sample size（N） | Statistical test | P values |
|---|---|---|---|
| 1C | Sham:<br>n = 16 mice<br>TBI:<br>n = 16 mice | Student t-test | Sham vs. TBI: P < 0.0001 **** |
| 1E | Sham:<br>n = 8 mice<br>TBI:<br>n = 8 mice<br>TNS:<br>n = 8 mice<br>TBI+TNS:<br>n = 8 mice | One-way ANOVA | Sham vs. TBI: P = 0.0004 ***<br>Sham vs. TNS: P = 0.7548 ns<br>TBI vs. TBI+TNS: P = 0.0021 **<br>TNS vs. TBI+TNS: P = 0.3804 ns |
| 1F | Sham:<br>n = 8 mice<br>TBI:<br>n = 8 mice<br>TNS:<br>n = 8 mice<br>TBI+TNS:<br>n = 8 mice | One-way ANOVA | Sham vs. TBI: P = 0.0050 **<br>Sham vs. TNS: P = 0.1607 ns<br>TBI vs. TBI+TNS: P = 0.0187 *<br>TNS vs. TBI+TNS: P = 0.0551 ns |
| 1H | Sham:<br>n = 8 mice<br>TBI:<br>n = 8 mice<br>TNS:<br>n = 8 mice<br>TBI+TNS:<br>n = 8 mice | One-way ANOVA | Sham vs. TBI: P < 0.0001 ****<br>Sham vs. TNS: P = 0.9387 ns<br>TBI vs. TBI+TNS: P = 0.0022 **<br>TNS vs. TBI+TNS: P = 0.2184 ns |
| 1I | Sham:<br>n = 8 mice<br>TBI:<br>n = 8 mice<br>TNS:<br>n = 8 mice<br>TBI+TNS:<br>n = 8 mice | One-way ANOVA | Sham vs. TBI: P < 0.0001 ****<br>Sham vs. TNS: P = 0.8863 ns<br>TBI vs. TBI+TNS: P = 0.0148 *<br>TNS vs. TBI+TNS: P = 0.0069 ** |
| 1K | Sham: | Student t- | Sham vs. TBI: P < 0.0001 **** |

| | | | |
|---|---|---|---|
| | n = 16 mice<br>TBI:<br>n = 16 mice | test | |
| 1L | Sham:<br>n = 8 mice<br>TBI:<br>n = 8 mice<br>TNS:<br>n = 8 mice<br>TBI+TNS:<br>n = 8 mice | One-way ANOVA | Sham vs. TBI: P < 0.0001 ****<br>Sham vs. TNS: P = 0.9997 ns<br>TBI vs. TBI+TNS: P = 0.0107 *<br>TNS vs. TBI+TNS: P < 0.0001 **** |
| 1M | Sham:<br>n = 8 mice<br>TBI:<br>n = 8 mice<br>TNS:<br>n = 8 mice<br>TBI+TNS:<br>n = 8 mice | One-way ANOVA | Sham vs. TBI: P < 0.0001 ****<br>Sham vs. TNS: P = 0.0421 *<br>TBI vs. TBI+TNS: P = 0.0005 ***<br>TNS vs. TBI+TNS: P < 0.0001 **** |
| 1N | Sham:<br>n = 8 mice<br>TBI:<br>n = 8 mice<br>TNS:<br>n = 8 mice<br>TBI+TNS:<br>n = 8 mice | One-way ANOVA | Sham vs. TBI: P = 0.0106 *<br>Sham vs. TNS: P = 0.5493 ns<br>TBI vs. TBI+TNS: P > 0.9999 ns<br>TNS vs. TBI+TNS: P = 0.2093 ns |
| 2B | Before:<br>n = 17 cells<br>After:<br>n = 17 cells | Two-way ANOVA | Before vs. After:<br>0 > 0.9999 ns<br>20 = 0.2255 ns<br>40 = 0.0899 ns<br>60 = 0.0307 *<br>80 = 0.0046 **<br>100 = 0.0052 ** |
| 2F | Saline / Ctrl:<br>n = 8 mice<br>Saline / hM3Dq:<br>n = 8 mice<br>CNO / Ctrl:<br>n = 8 mice<br>CNO / hM3Dq:<br>n = 8 mice | Student t-test | Saline / Ctrl vs. Saline / hM3Dq:<br>P = 0.9792 ns<br>CNO / Ctrl vs. CNO / hM3Dq:<br>P = 0.0014 ** |
| 2G | Saline / Ctrl:<br>n = 8 mice | Student t-test | Saline / Ctrl vs. Saline / hM3Dq:<br>P = 0.9999 ns |

| | | | |
|---|---|---|---|
| | Saline / hM3Dq:<br>n = 8 mice<br>CNO / Ctrl:<br>n = 8 mice<br>CNO / hM3Dq:<br>n = 8 mice | | CNO / Ctrl vs. CNO / hM3Dq:<br>P = 0.0126 * |
| 2H | Saline / Ctrl:<br>n = 8 mice<br>Saline / hM3Dq:<br>n = 8 mice<br>CNO / Ctrl:<br>n = 8 mice<br>CNO / hM3Dq:<br>n = 8 mice | Student t-test | Saline / Ctrl vs. Saline / hM3Dq:<br>P = 0.8298 ns<br>CNO / Ctrl vs. CNO / hM3Dq:<br>P = 0.0105 * |
| 2I | Saline / Ctrl:<br>n = 8 mice<br>Saline / hM3Dq:<br>n = 8 mice<br>CNO / Ctrl:<br>n = 8 mice<br>CNO / hM3Dq:<br>n = 8 mice | Student t-test | Saline / Ctrl vs. Saline / hM3Dq:<br>P = 0.9887 ns<br>CNO / Ctrl vs. CNO / hM3Dq:<br>P = 0.0002 *** |
| 2J | Saline / Ctrl:<br>n = 8 mice<br>Saline / hM3Dq:<br>n = 8 mice<br>CNO / Ctrl:<br>n = 8 mice<br>CNO / hM3Dq:<br>n = 8 mice | Student t-test | Saline / Ctrl vs. Saline / hM3Dq:<br>P = 0.8169 ns<br>CNO / Ctrl vs. CNO / hM3Dq:<br>P = 0.0007 *** |
| 2K | Saline / Ctrl:<br>n = 8 mice<br>Saline / hM3Dq:<br>n = 8 mice<br>CNO / Ctrl:<br>n = 8 mice<br>CNO / hM3Dq:<br>n = 8 mice | Student t-test | Saline / Ctrl vs. Saline / hM3Dq:<br>P = 0.8211 ns<br>CNO / Ctrl vs. CNO / hM3Dq:<br>P = 0.0013 ** |
| 2L | Saline / Ctrl:<br>n = 8 mice<br>Saline / hM3Dq:<br>n = 8 mice<br>CNO / Ctrl:<br>n = 8 mice | Student t-test | Saline / Ctrl vs. Saline / hM3Dq:<br>P = 0.9667 ns<br>CNO / Ctrl vs. CNO / hM3Dq:<br>P = 0.0091 ** |

| | | | |
|---|---|---|---|
| | CNO / hM3Dq:<br>n = 8 mice | | |
| 2M | Saline / Ctrl:<br>n = 8 mice<br>Saline / hM3Dq:<br>n = 8 mice<br>CNO / Ctrl:<br>n = 8 mice<br>CNO / hM3Dq:<br>n = 8 mice | Student t-test | Saline / Ctrl vs. Saline / hM3Dq:<br>P = 0.8405 ns<br>CNO / Ctrl vs. CNO / hM3Dq:<br>P = 0.0071 ** |
| 2N | Saline / Ctrl:<br>n = 8 mice<br>Saline / hM3Dq:<br>n = 8 mice<br>CNO / Ctrl:<br>n = 8 mice<br>CNO / hM3Dq:<br>n = 8 mice | Student t-test | Saline / Ctrl vs. Saline / hM3Dq:<br>P = 0.5700 ns<br>CNO / Ctrl vs. CNO / hM3Dq:<br>P = 0.0145 * |
| 2O | Saline / Ctrl:<br>n = 8 mice<br>Saline / hM3Dq:<br>n = 8 mice<br>CNO / Ctrl:<br>n = 8 mice<br>CNO / hM3Dq:<br>n = 8 mice | Student t-test | Saline / Ctrl vs. Saline / hM3Dq:<br>P = 0.1865 ns<br>CNO / Ctrl vs. CNO / hM3Dq:<br>P = 0.0021 ** |
| 2P | Saline / Ctrl:<br>n = 8 mice<br>Saline / hM3Dq:<br>n = 8 mice<br>CNO / Ctrl:<br>n = 8 mice<br>CNO / hM3Dq:<br>n = 8 mice | Student t-test | Saline / Ctrl vs. Saline / hM3Dq:<br>P = 0.6412 ns<br>CNO / Ctrl vs. CNO / hM3Dq:<br>P = 0.8806 ns |
| 2Q | Saline / Ctrl:<br>n = 8 mice<br>Saline / hM3Dq:<br>n = 8 mice<br>CNO / Ctrl:<br>n = 8 mice<br>CNO / hM3Dq:<br>n = 8 mice | Student t-test | Saline / Ctrl vs. Saline / hM3Dq:<br>P = 0.9766 ns<br>CNO / Ctrl vs. CNO / hM3Dq:<br>P = 0.3278 ns |
| S3B | Saline / Ctrl:<br>n = 8 mice | Student t-test | Saline / Ctrl vs. Saline / hM3Dq:<br>P = 0.8919 ns |

| | | | |
|---|---|---|---|
| | Saline / hM3Dq:<br>n = 8 mice<br>CNO / Ctrl:<br>n = 8 mice<br>CNO / hM3Dq:<br>n = 8 mice | | CNO / Ctrl vs. CNO / hM3Dq:<br>P = 0.9855 ns |
| S3C | Saline / Ctrl:<br>n = 8 mice<br>Saline / hM3Dq:<br>n = 8 mice<br>CNO / Ctrl:<br>n = 8 mice<br>CNO / hM3Dq:<br>n = 8 mice | Student t-test | Saline / Ctrl vs. Saline / hM3Dq:<br>P = 0.9471 ns<br>CNO / Ctrl vs. CNO / hM3Dq:<br>P = 0.7152 ns |
| S3D | Saline / Ctrl:<br>n = 8 mice<br>Saline / hM3Dq:<br>n = 8 mice<br>CNO / Ctrl:<br>n = 8 mice<br>CNO / hM3Dq:<br>n = 8 mice | Student t-test | Saline / Ctrl vs. Saline / hM3Dq:<br>P = 0.9311 ns<br>CNO / Ctrl vs. CNO / hM3Dq:<br>P = 0.9077 ns |
| S3E | Saline / Ctrl:<br>n = 8 mice<br>Saline / hM3Dq:<br>n = 8 mice<br>CNO / Ctrl:<br>n = 8 mice<br>CNO / hM3Dq:<br>n = 8 mice | Student t-test | Saline / Ctrl vs. Saline / hM3Dq:<br>P = 0.8439 ns<br>CNO / Ctrl vs. CNO / hM3Dq:<br>P = 0.8421 ns |
| S3F | Saline / Ctrl:<br>n = 8 mice<br>Saline / hM3Dq:<br>n = 8 mice<br>CNO / Ctrl:<br>n = 8 mice<br>CNO / hM3Dq:<br>n = 8 mice | Student t-test | Saline / Ctrl vs. Saline / hM3Dq:<br>P = 0.8903 ns<br>CNO / Ctrl vs. CNO / hM3Dq:<br>P = 0.8839 ns |
| S3G | Saline / Ctrl:<br>n = 8 mice<br>Saline / hM3Dq:<br>n = 8 mice<br>CNO / Ctrl:<br>n = 8 mice | Student t-test | Saline / Ctrl vs. Saline / hM3Dq:<br>P = 0.5904 ns<br>CNO / Ctrl vs. CNO / hM3Dq:<br>P = 0.9023 ns |

| | | | |
|---|---|---|---|
| | CNO / hM3Dq:<br>n = 8 mice | | |
| S3H | Saline / Ctrl:<br>n = 8 mice<br>Saline / hM3Dq:<br>n = 8 mice<br>CNO / Ctrl:<br>n = 8 mice<br>CNO / hM3Dq:<br>n = 8 mice | Student t-test | Saline / Ctrl vs. Saline / hM3Dq:<br>P = 0.8714 ns<br>CNO / Ctrl vs. CNO / hM3Dq:<br>P = 0.9891 ns |
| S3I | Saline / Ctrl:<br>n = 8 mice<br>Saline / hM3Dq:<br>n = 8 mice<br>CNO / Ctrl:<br>n = 8 mice<br>CNO / hM3Dq:<br>n = 8 mice | Student t-test | Saline / Ctrl vs. Saline / hM3Dq:<br>P = 0.9762 ns<br>CNO / Ctrl vs. CNO / hM3Dq:<br>P = 0.9995 ns |
| 3D | Saline / Ctrl:<br>n = 8 mice<br>Saline / hM3Dq:<br>n = 8 mice<br>CNO / Ctrl:<br>n = 8 mice<br>CNO / hM3Dq:<br>n = 8 mice | Student t-test | Saline / Ctrl vs. Saline / hM3Dq:<br>P = 0.7526 ns<br>CNO / Ctrl vs. CNO / hM3Dq:<br>P = 0.0112 * |
| 3E | Saline / Ctrl:<br>n = 8 mice<br>Saline / hM3Dq:<br>n = 8 mice<br>CNO / Ctrl:<br>n = 8 mice<br>CNO / hM3Dq:<br>n = 8 mice | Student t-test | Saline / Ctrl vs. Saline / hM3Dq:<br>P = 0.9998 ns<br>CNO / Ctrl vs. CNO / hM3Dq:<br>P = 0.0097 ** |
| 3J | Ctrl:<br>n = 8 mice<br>taCasp3:<br>n = 8 mice | Student t-test | Ctrl vs. taCasp3: P = 0.0012 ** |
| 3K | Ctrl:<br>n = 8 mice<br>taCasp3:<br>n = 8 mice | Student t-test | Ctrl vs. taCasp3: P = 0.0045 ** |
| 3L | Ctrl:<br>n = 8 mice | Student t-test | Ctrl vs. taCasp3: P = 0.0031 ** |

| | | | |
|---|---|---|---|
| | taCasp3:<br>n = 8 mice | | |
| 3M | Ctrl:<br>n = 8 mice<br>taCasp3:<br>n = 8 mice | Student t-test | Ctrl vs. taCasp3: P = 0.7573 ns |
| 3N | Ctrl:<br>n = 8 mice<br>taCasp3:<br>n = 8 mice | Student t-test | Ctrl vs. taCasp3: P = 0.4370 ns |
| 4I | TBI<br>n = 4 mice<br>TBI+TNS:<br>n = 4 mice | Two-way ANOVA | TBI vs. TBI+TNS:<br>10°/s > 0.9999 ns<br>20°/s = 0.9997 ns<br>40°/s > 0.9999 ns<br>60°/s > 0.9999 ns<br>80°/s > 0.9999 ns<br>120°/s = 0.9897 ns<br>160°/s = 0.9987 ns<br>320°/s = 0.9991 ns |
| 4J | TBI<br>n = 4 mice<br>TBI+TNS:<br>n = 4 mice | Two-way ANOVA | TBI vs. TBI+TNS:<br>10°/s = 0.8490 ns<br>20°/s = 0.0192 *<br>40°/s < 0.0001 ****<br>60°/s < 0.0001 ****<br>80°/s < 0.0001 ****<br>120°/s < 0.0001 ****<br>160°/s < 0.0001 ****<br>320°/s = 0.0384 * |
| 4K | TBI<br>n = 4 mice<br>TBI+TNS:<br>n = 4 mice | Two-way ANOVA | TBI vs. TBI+TNS:<br>10°/s = 0.1995 ns<br>20°/s = 0.0021 **<br>40°/s < 0.0001 ****<br>60°/s < 0.0001 ****<br>80°/s < 0.0001 ****<br>120°/s < 0.0001 ****<br>160°/s < 0.0001 ****<br>320°/s < 0.0001 **** |
| 4L | TBI<br>n = 4 mice<br>TBI+TNS:<br>n = 4 mice | Two-way ANOVA | TBI vs. TBI+TNS:<br>10°/s = 0.9200 ns<br>20°/s = 0.8806 ns<br>40°/s = 0.6140 ns<br>60°/s = 0.8250 ns |

| | | | |
|---|---|---|---|
| | | | 80°/s = 0.1125 ns<br>120°/s = 0.3883 ns<br>160°/s = 0.4057 ns<br>320°/s = 0.1194 ns |
| 4O | Ctrl:<br>n = 4 mice<br>hM3Dq:<br>n = 4 mice | Two-way ANOVA | Ctrl vs. hM3Dq:<br>10°/s = 0.9959 ns<br>20°/s = 0.9993 ns<br>40°/s = 0.9953 ns<br>60°/s = 0.0823 ns<br>80°/s = 0.9947 ns<br>120°/s = 0.9651 ns<br>160°/s = 0.9967 ns<br>320°/s = 0.9994 ns |
| 4P | Ctrl:<br>n = 4 mice<br>hM3Dq:<br>n = 4 mice | Two-way ANOVA | Ctrl vs. hM3Dq:<br>10°/s = 0.9859 ns<br>20°/s = 0.1321 ns<br>40°/s = 0.0044 **<br>60°/s < 0.0001 ****<br>80°/s < 0.0001 ****<br>120°/s < 0.0001 ****<br>160°/s = 0.0040 **<br>320°/s = 0.0407 * |
| 4Q | Ctrl:<br>n = 4 mice<br>hM3Dq:<br>n = 4 mice | Two-way ANOVA | Ctrl vs. hM3Dq:<br>10°/s = 0.9973 ns<br>20°/s = 0.5626 ns<br>40°/s = 0.0976 ns<br>60°/s = 0.0006 ***<br>80°/s < 0.0001 ****<br>120°/s < 0.0001 ****<br>160°/s = 0.0092 **<br>320°/s = 0.2669 ns |
| 4R | Ctrl:<br>n = 4 mice<br>hM3Dq:<br>n = 4 mice | Two-way ANOVA | Ctrl vs. hM3Dq:<br>10°/s = 0.9964 ns<br>20°/s > 0.9999 ns<br>40°/s > 0.9999 ns<br>60°/s = 0.9793 ns<br>80°/s = 0.7364 ns<br>120°/s = 0.3403 ns<br>160°/s = 0.3165 ns<br>320°/s = 0.7128 ns |
| 5M | 1 mW n=20,<br>2 mW n=20, | One-way ANOVA | 1 mW vs. 2 mW: P = 0.0236 *<br>2 mW vs. 3 mW: P < 0.0001 **** |

| | | | |
|---|---|---|---|
| | 3 mW n=20,<br>4 mW n=20,<br>2 mW+YO n=20 | | 3 mW vs. 4 mW: P = 0.0109 *<br>2 mW vs. 2 mW+YO: P < 0.0001 **** |
| 6I | Saline / Ctrl:<br>n = 8 mice<br>Saline / hM3Dq:<br>n = 8 mice<br>CNO / Ctrl:<br>n = 8 mice<br>CNO / hM3Dq:<br>n = 8 mice | Student t-test | Saline / Ctrl vs. Saline / hM3Dq:<br>P = 0.9854 ns<br>CNO / Ctrl vs. CNO / hM3Dq:<br>P = 0.0463 * |
| 6J | Saline / Ctrl:<br>n = 8 mice<br>Saline / hM3Dq:<br>n = 8 mice<br>CNO / Ctrl:<br>n = 8 mice<br>CNO / hM3Dq:<br>n = 8 mice | Student t-test | Saline / Ctrl vs. Saline / hM3Dq:<br>P = 0.9946 ns<br>CNO / Ctrl vs. CNO / hM3Dq:<br>P = 0.0002 *** |
| 6M | Ctrl:<br>n = 8 mice<br>taCasp3:<br>n = 8 mice | Student t-test | Ctrl vs. taCasp3: P = 0.0002 *** |
| 6N | Ctrl:<br>n = 8 mice<br>taCasp3:<br>n = 8 mice | Student t-test | Ctrl vs. taCasp3: P = 0.0021 ** |
| 6O | Ctrl:<br>n = 8 mice<br>taCasp3:<br>n = 8 mice | Student t-test | Ctrl vs. taCasp3: P = 0.0166 * |
| 6P | Ctrl:<br>n = 8 mice<br>taCasp3:<br>n = 8 mice | Student t-test | Ctrl vs. taCasp3: P = 0.0001 *** |
| 6Q | Ctrl:<br>n = 8 mice<br>taCasp3:<br>n = 8 mice | Student t-test | Ctrl vs. taCasp3: P = 0.0002 *** |
| 7K | 1 mW n=20,<br>2 mW n=20,<br>3 mW n=20,<br>2 mW+Haldol 0.4 mg/kg n=20, | One-way ANOVA | 1 mW vs. 2 mW: P < 0.0001 ****<br>2 mW vs. 3 mW: P < 0.0001 ****<br>2 mW vs. 2 mW+Haldol 0.4 mg/kg:<br>P < 0.0001 **** |

| | | | |
|---|---|---|---|
| | 2 mW+Haldol 1.2 mg/kg n=20 | | 2 mW vs. 2 mW+Haldol 1.2 mg/kg: P < 0.0001 **** |
